# Active Vision in Sight Recovery Individuals with a History of Long-Lasting Congenital Blindness

**DOI:** 10.1523/ENEURO.0051-22.2022

**Published:** 2022-09-29

**Authors:** José P. Ossandón, Paul Zerr, Idris Shareef, Ramesh Kekunnaya, Brigitte Röder

**Affiliations:** 1 Biological Psychology and Neuropsychology, Hamburg University, 20146 Hamburg, Germany; 2 Experimental Psychology, Helmholtz Institute, Utrecht University, 3584 CS, Utrecht, The Netherlands; 3 Child Sight Institute, Jasti V Ramanamma Children's Eye Care Center, LV Prasad Eye Institute, 500034 Hyderabad, India

**Keywords:** congenital cataracts, eye movements, nystagmus, sensitive period, sight restoration

## Abstract

What we see is intimately linked to how we actively and systematically explore the world through eye movements. However, it is unknown to what degree visual experience during early development is necessary for such systematic visual exploration to emerge. The present study investigated visual exploration behavior in 10 human participants whose sight had been restored only in childhood or adulthood, after a period of congenital blindness because of dense bilateral congenital cataracts. Participants freely explored real-world images while their eye movements were recorded. Despite severe residual visual impairments and gaze instability (nystagmus), visual exploration patterns were preserved in individuals with reversed congenital cataract. Modeling analyses indicated that, similar to healthy control subjects, visual exploration in individuals with reversed congenital cataract was based on the low-level (luminance contrast) and high-level (object components) visual content of the images. Moreover, participants used visual short-term memory representations for narrowing down the exploration space. More systematic visual exploration in individuals with reversed congenital cataract was associated with better object recognition, suggesting that active vision might be a driving force for visual system development and recovery. The present results argue against a sensitive period for the development of neural mechanisms associated with visual exploration.

## Significance Statement

Humans explore the visual world with systematic patterns of eye movements, but it is unknown whether early visual experience is necessary for the acquisition of visual exploration. Here, we show that sight recovery individuals who had been born blind demonstrate highly systematic eye movements while exploring real-world images, despite visual impairments and pervasive gaze instability. In fact, their eye movement patterns were predicted by those of normally sighted control subjects and models calculating eye movements based on low-level and high-level visual features and, moreover, taking memory information into account. Since object recognition performance was associated with systematic visual exploration, it was concluded that eye movements might be a driving factor for the development of the visual system.

## Introduction

Prolonged visual deprivation from birth has been observed to result in the irreversible impairment of several visual functions (Lewis and Maurer, 2005; Röder and Kekunnaya, 2021). These findings have been taken as evidence for “sensitive periods” in brain development, defined as epochs during which adequate input is essential for full functional development (Knudsen, 2004; Hensch, 2005). In humans, sensitive periods have been studied in individuals who had been born blind or with severe visual impairments because of dense, bilateral cataracts and who later received cataract-removal surgery at different times during infancy, childhood, or even adulthood (Maurer et al., 2007; Röder et al., 2013; Ganesh et al., 2014). Despite improvements in vision after congenital cataract removal (Wright et al., 1992), basic visual abilities such as visual acuity (Ellemberg et al., 1999; Lambert et al., 2006) remain permanently impaired, especially if cataracts are not treated within the first few weeks of life. Moreover, higher-order visual functions such as feature binding and within-category viewpoint-independent discrimination, particularly of faces, have been found to only partially recover after congenital cataract surgery, and not to the degree expected by the recovery of visual acuity (Le Grand et al., 2001; Putzar et al., 2007, 2010; Ostrovsky et al., 2009). In addition to these perceptual deficits, individuals who had prolonged congenital bilateral visual deprivation (>8 weeks) typically also experience nystagmus (Rogers et al., 1981; Lambert et al., 2006; Birch et al., 2009). Nystagmus is a disorder of gaze stability that results in continuous, periodic, and involuntary motion of the eyes.

It has recently been shown that despite some distortions because of the superimposed nystagmus, eye movements to simple visual stimuli were reasonably precise and fast in individuals with reversed congenital cataract (Zerr et al., 2020). However, it is unclear whether higher levels of ocular control, such as the ability to generate typical patterns of active visual exploration of natural stimuli, recover after a transient phase of congenital visual deprivation. Active visual exploration is crucial for visual functions such as visual search and object identification, especially in noisy or ambiguous conditions (Einhäuser et al., 2004; Holm et al., 2008; Kietzmann et al., 2011). Furthermore, active visual exploration has been shown to be relevant for visual memory formation in typically sighted individuals (Hannula, 2010).

Previous research has suggested that visual exploration is guided by both bottom-up (stimulus-driven) and top-down mechanisms, which jointly define the direction toward which the eyes move. Stimulus-driven mechanisms use input characteristics such as luminance, color, orientation, and motion (Veale et al., 2017), whereas top-down mechanisms consider goals, memory, and contextual factors (Eckstein, 2011; Tatler et al., 2011; König et al., 2016). Stimulus-driven “saliency” models have successfully used low-level and high-level visual features to predict human eye movements during free viewing of scenes (Itti and Koch, 2000; Tatler et al., 2005; Kümmerer et al., 2017). Additionally, the repeated presentation of the same image has been used to assess the effects of short-term memory on visual exploration, that is, a nonreflexive aspect of gaze control (Ryan et al., 2000; Smith et al., 2006; Kaspar and König, 2011a,b). If an image is repeatedly encountered, the spread of visual exploration decreases (Hannula, 2010). It has been hypothesized that short-term memory representations provide top-down information, which, combined with bottom-up stimulus-driven maps in so-called priority maps, guide eye movements (Veale et al., 2017).

The degree to which the development of bottom-up and top-down mechanisms of active visual exploration depend on typical visual input after birth is unknown. Theories from developmental psychology have suggested that active visual exploration in infants is instrumental for the development of object knowledge (Johnson and Johnson, 2000). It remains to be investigated whether visual recovery after late sight restoration affects bottom-up, stimulus-driven visual exploration (Einhäuser et al., 2008a; Açık et al., 2009; Nuthmann and Henderson, 2010), and/or top-down, for instance, memory-based, visual exploration (Hannula, 2010).

In the present study, we used a free-viewing task in a sample of 10 individuals who had been born with dense, bilateral cataracts, which had been surgically removed later in life [congenital cataract reversal (CC) group; Table 1] in some participants, only in late childhood or adulthood. The distribution of gazed locations elicited by photographic stimuli (close-up images of different objects, plants, animals, and buildings) were assessed and compared with the typical visual exploration patterns of age-matched, normally sighted controls [sighted control (SC) group]. Further, the CC group was compared with individuals with nystagmus because of reasons other than congenital cataracts [nystagmus control (NC) group], and individuals with a history of developmental cataracts [developmental cataract reversal (DC) group], to isolate group differences specific to early visual deprivation rather than a general history of visual deficits. Finally, to explore top-down influences on visual exploration, the effects of short-term memory on eye movements were assessed by assessing the adaptation of visual exploration patterns for images which were repeatedly presented.

**Table 1:** Participants description

	Age at testing (years)	Age at surgery (months)	Cataract type at surgery	Presurgical visual acuity (best eye)	Most recent visual acuity (CC/DC: postsurgical; best eye)		Cataract family history
					Decimal	logMAR	
CC group (*N* = 10)							
CC-1	16.9	4	Dense	FFL+	0.33	0.47	No
CC-2	13.5	83	Absorbed	CF 3 m	0.15	0.79	No
CC-3	31.7	168	Absorbed	Unknown	0.1	1	Yes
CC-4	42.9	264	Absorbed	0.06 (decimal)	0.16	0.79	Yes
CC-5	16.1	186	Absorbed	0.03 (decimal)	0.08	1.1	No
CC-6	12.3	138	Dense	CF CF	0.03	1.4	Yes
CC-7	21.7	213	Dense	CF 0.5 m	0.12	0.9	Yes
CC-8	10.7	17	Dense	PL at 0.5 m	0.25	0.6	No
CC-9	23.5	3	Dense	FFL–	0.25	0.6	No
CC-10	17.2	34	Dense	FFL+	0.16	0.79	No
Summary	M: 20.7 R: 10–42	M: 111 (9.2 years) R: 4–264			GM: 0.14 R: 0.03–0.3	M: 0.86 R: 0.47–1.4	
DC group (*N* = 9)							
DC-1	24.4	31	Not dense	FFL+	0.5	0.3	No
DC-2	13.8	89	Dense	CF 1 m	0.66	0.17	Unknown
DC-3	16.2	130	Not dense	0.4	0.8	0.09	No
DC-4	13.2	71	Dense	FFL+	0.46	0.33	Unknown
DC-5	17.3	91	Not dense	0.16	0.8	0.09	Unknown
DC-6	11.5	91	Not dense	0.25	0.8	0.09	Unknown
DC-7	18.8	208	Not dense	0.2	0.7	0.13	No
DC-8	11.6	54	Dense	CF 1 m	1	0	Unknown
DC-9	13.5	30	Dense	FFL+	0.66	0.17	Unknown
Summary	M:15.6 R:11–24	M: 88.3 (7.4 years) R: 30–208	4 dense		GM: 0.7 R: 0.46 – 1	M: 0.16 R: 0 – 0.33	
NC group (*N* = 10)							
Summary	M: 15 R: 8–37				GM: 0.45 R: 0.25–0.8	M: 0.35 R: 0.1–0.6	
SC group (*N* = 13)							
Summary	M: 23.7 R: 11–40				1 (all)	0 (all)	

PL, Perception light; CF, counting finger (equivalence with logMAR acuity has been reported to be 1.7–2.0 at 30 cm; Schulze-Bonsel et al., 2006); CF CF, counting finger close to face; FFL, fixate and follows light; M, mean; GM, geometric mean; R, range.

10.1523/ENEURO.0051-22.2022.tab1-1 Table 1-1 The relationship between visual acuity and age of surgery (CC and DC groups) and age of testing (all groups). Download Table 1-1, EPS file.

## Materials and Methods

### Participants

A total of 42 participants from four different populations were recruited at the LV Prasad Eye Institute and the local community of Hyderabad (India).

#### Congenital cataract reversal individuals (CC group)

Individuals were selected from a large number of patients who had been treated with the diagnosis of congenital cataracts. Based on medical records, a clinical history of bilateral congenital cataracts and a history of patterned visual deprivation were confirmed. A lack of fundus view and a lack of retinal glow were considered as evidence for the absence of patterned input reaching the retina before cataract surgery. Additionally, the presence of nystagmus, sensory strabismus, positive family history as well as absorbed lenses aided in the classification of CC participants.

The CC group consisted of 10 participants (2 females; mean age, 20.7 years; age range, 10.7–42.9 years) who had received cataract removal surgery at a mean age of 9.2 years (age range, 3 months to 22 years). These individuals were tested on average 11.4 years after cataract removal surgery (range, 7 months to 23.2 years). Of the 10 participants, 5 had a documented history of strabismus (esotropia, 2 participants; exotropia, 3 participants), 7 had implanted intraocular lenses, and the remaining 3 used corrective glasses. Four CC individuals had a documented family history of congenital cataracts, and four CC individuals had absorbed cataracts when presenting at the LV Prasad Eye Institute. Absorption of cataracts in middle to late childhood has been regularly observed in individuals born with dense congenital cataracts. Absorbed cataracts can be unambiguously differentiated from nondense or partial cataracts by, for instance, the morphology of the lens, anterior capsule wrinkling, as well as plaque or thickness of the stroma. Absorbed cataracts strongly imply dense cataracts, and therefore blindness, at birth. Presurgical visual acuity measurements in severely visually deprived individuals confirmed that at least 7 of 10 CC individuals were blind (i.e., had a visual acuity of <3/60; World Health Organization, 2019). The remaining three CC individuals had absorbed lenses; their presurgery vison corresponded to severe visual impairment, as defined by the World Health Organization. All CC participants additionally had nystagmus, which is strong evidence for the absence of patterned vision at birth. CC participants’ postsurgical visual acuity of the better eye ranged from 0.03 to 0.33 decimal units [geometric mean, 0.14; logarithm of the minimum angle of resolution (logMar), 0.47–1.4; logMar mean, 0.86]. A detailed description of CC participants is presented in Table 1 (see Schulze-Bonsel et al., 2006 for visual acuity equivalences and Extended Data Table 1-1).

#### Developmental cataract reversal group (DC group)

This control group allowed us to estimate the role of vision at birth for the acquisition of visual exploration behavior. The DC group consisted of nine individuals (four females; mean age, 15.6 years; age range, 11.6–24.4) with a history of bilateral cataracts, but not dense and/or congenital cataracts. These individuals allowed us to control for task-independent effects on eye movements because of cataract surgery (e.g., exploring the images with intraocular lenses). Cataract removal surgery had been performed at a mean age of 7.4 years (age range, 2.8–17.3 years); they were tested on average 8.2 years (range, 1.5–21.8 years) postsurgery. DC participants’ postsurgical visual acuity ranged from 0.46 to 1 decimal units (geometrical mean, 0.7; logMar, 0–0.33; logMar mean, 0.16). All DC participants were fitted with intraocular lenses.

Retrospective classification of CC and DC participants comes with some degree of uncertainty. However, the use of the classification criteria as implemented in the present study have recently been confirmed by an electrophysiological biomarker (Sourav et al., 2020).

#### Nystagmus group (NC group)

To disentangle the effects of congenital visual deprivation from the effects of prevailing sensory nystagmus, which was present in all CC participants, individuals with nystagmus because of conditions other than congenital cataracts were tested as additional control subjects. Individuals in this group did not experience a phase of severe visual deprivation. Therefore, this group allowed us to distinguish which changes in visual exploration behavior can be attributed to the effects of nystagmus versus congenital visual deprivation. This group comprised 10 participants (1 female; mean age, 15.0 years; age range, 8.7–37.3 years) with infantile nystagmus syndrome (idiopathic, 9 participants; oculocutaneous albinism, 1 participant), without a history of cataracts, severe visual impairment, or blindness. NC participants’ visual acuity ranged from 0.25 to 0.8 decimal units (geometrical mean, 0.45; logMar, 0.1–0.6; logMar mean, 0.35).

#### The sighted control group (SC group)

The SC group consisted of 13 individuals (3 females; mean age, 23.7 years; age range, 11.2–40.6 years) with normal or corrected-to-normal vision. This group was partially age matched to the CC group (no significant difference in age at testing; *t*_(21)_ = −0.84, *p* = 0.41). The SC group allowed us to establish typical eye movement parameters for healthy individuals in the current experimental setting and for the used images.

All individuals were tested at the LV Prasad Eye Institute. None of the participants had any other sensory deficit or neurologic disorder, diagnosed or self-reported. Expenses associated with taking part in the study were reimbursed. Minors additionally received a small present. Participants, and if applicable, their legal guardians, were informed about the study and received the instructions in one of the languages they were able to understand (in most cases Telegu, Hindi, or English). All participants gave written informed consent before participating in the study; in the case of minors, legal guardians additionally provided informed consent. The study was approved by the ethics board of the Faculty of Psychology and Human Movement Science of the University of Hamburg (Germany) and by the ethics board of the LV Prasad Eye Institute.

### Stimuli

Forty-nine images from the Natural Face and Object Stimuli image set were used in this study (Rossion et al., 2015; https://face-categorization-lab.webnode.com/resources/natural-face-stimuli/). The images displayed objects representing seven different categories: animals, chairs, fruits, guitars, houses, plants, and telephones. Objects were located close to the center of the image. Images were displayed in grayscale at an 800 × 800 pixel resolution, subtending 19.6 × 19.6 visual degrees. Stimuli were generated in MATLAB (MathWorks) using Psychtoolbox 3 (Brainard, 1997; Kleiner et al., 2007) on a Windows 7 PC and presented with a 24 inch Eizo FG2421 LCD monitor at a resolution of 1920 × 1080 at 120 Hz.

Because of copyright concerns, the figures shown here use line drawings of the actual images presented during the experiment.

### Eye-tracking and calibration

Eye movements were recorded with a video-based binocular eye-tracking system at 500 Hz (EyeLink1000 Plus, SR Research). Subjects were seated in a darkened room and placed their heads on a chin rest such that their eyes were at a distance of 60 cm from the screen. Because of several participants presenting with nystagmus, it was not possible to use the built-in standard online calibration method of the eye-tracker system. Instead, a custom-made calibration routine was used. This calibration routine used five screen positions as points (presented at the screen center, 15° right and left of the center and 8.5° above and below the center). Each participant was asked to look at these five points. Next, the screen center position was displayed again, to estimate calibration error. The experimenter manually controlled the calibration. During the presentation of each calibration position, the experimenter decided whether an eye movement was performed to the corresponding point and selected low-velocity periods of the nystagmus at each calibration point. These low-velocity periods typically follow the corrective saccade of the nystagmus; that is, they are aligned with the target position. The online calibration was performed to visually confirm that calibration points aligned with the five position patterns. This confirmation was necessary to decide whether the procedure had to be repeated, or if the calibration was sufficiently precise to continue. During offline calibration, low-velocity periods of nystagmus were selected as described for online calibration.

The median positions of the selected gaze samples were fitted with a polynomial function (Stampe, 1993) to the corresponding screen positions. This is the same algorithm as the one implemented in the EyeLink eye-tracker software. The same calibration procedure was applied to all participants regardless of whether they had nystagmus or not. Calibration error was calculated only for the central position of the screen and did not differ between groups (robust linear model contrasts; all contrasts, *p *>* *0.05; mean CC group: 0.83°; SD, 0.95; mean SC group: 0.31°; SD, 0.12; mean DC group: 0.59°; SD, 0.37; mean NC group: 0.78°; SD, 0.83).

A certain proportion of gaze data was missing when the gaze fell outside of the image or during periods when the eye tracker lost the pupil. On an average, 9.9% of CC participants’ gaze data was missing, compared with 3.69% for SC, 4.27% for DC, and 9.93% for NC participants.

To guarantee a sufficiently reliable estimate, only the visual exploration data from images with at least 50% valid recordings (i.e., gaze location values within the image) were included for analyses. Under this criterion, seven, one, one, and six image explorations had to be disregarded for the CC, SC, DC, and NC group, respectively. In total, we discarded <0.6% of the data.

### Procedure

After the calibration was completed, the experiment was conducted in two blocks of 14 trials each. A trial consisted of two images presented sequentially. Each trial started with a white fixation dot (diameter, ∼1.8°) presented in the center of the screen for 1 s, followed by the presentation of the first image for 4 s. Next, a central white fixation dot was shown a second time for 1 s, which was followed by the presentation of the second image for 4 s. Participants were instructed to visually explore the images and report the names of the objects they had encountered in the two images at the end of the trial. After participants provided the names of the two images, the experimenter decided whether each image was correctly named. The experimenters knew about the possible categories and were instructed to accept responses at the exemplar level (e.g., banana) and categorical level (e.g., fruit).

In 7 of the 28 trial image pairs, the same image was presented as the first and second image. In another seven trial pairs, the two images were different, but from the same object category. In the remaining 14 trial pairs, the images were from different categories. Image presentation order was randomized across subjects with respect to pair type (repeated image, repeated category, different category). Order of presentation within a pair in the repeated and different category pairs was randomized across subjects. The experiment took ∼15–20 min, depending on the duration of the eye-tracker calibration procedure.

### Data analysis and statistics

The common procedure in eye-tracking research is to use fixation positions as the unit of analysis. However, the nystagmus of the CC and NC individuals made it impossible to define fixations by using typical velocity and acceleration thresholds. Hence, the dependent variables for all participants were calculated with respect to the position of all eye-tracking data samples (subsequently referred to as “gaze” data, obtained at a 500 Hz sampling rate). Several studies have shown that individuals with nystagmus gather information during the complete period of the nystagmus, and not only during the low-velocity phase (Jin et al., 1989; Goldstein et al., 1992; Waugh and Bedell, 1992; Dunn et al., 2014). Therefore, for uniformity of the analysis across groups, all gaze data were used, including high-velocity samples that would normally be considered saccades.

#### Instantaneous gaze velocity

The extent of gaze instability in all participants was estimated by deriving “instantaneous gaze velocity.” Usually, gaze velocity is calculated from multiple samples to remove high-frequency noise inherent to oculomotor recordings (Stampe, 1993; Holmqvist, 2011). In the present study, we used a modified version of the 2-point central difference algorithm (Bahill et al., 1982) that is standard in the literature (Engbert and Kliegl, 2003; Dimigen et al., 2009; Otero-Millan et al., 2014), and used as the default by the EyeLink eye-tracker (SR Research, 2019). For a sample point *n*, the corresponding instantaneous gaze velocity [in visual degrees per second (°/s)], is defined as the sum of six nonconsecutive eye-tracking gaze samples (position in screen pixels), as follows:

$${\text{Velocity}}_{\left(n\right)}=\frac{SR\times ({\text{gaze}}_{\left(n+4\right)}+{\text{gaze}}_{\left(n+3\right)}+{\text{gaze}}_{\left(n+2\right)}-{\text{gaze}}_{\left(n-2\right)}-{\text{gaze}}_{\left(n-3\right)}-{\text{gaze}}_{\left(n-4\right)})}{18\times \text{PPD}},$$

where SR is the eye-tracker sampling rate (500 Hz), PPD is the pixels-per-degree resolution (∼40.6), and 18 corresponds to the sum of the number of samples in the intervals used in the calculation [ (4−–4)+(3−–3)+(2−–2)]. This calculation was performed separately for the horizontal and vertical eye movement components.

#### Entropy

To assess the spread or dispersion of visual exploration, the informational entropy of the spatial distribution of gazed locations was calculated for each image and participant (Açık et al., 2010; Wilming et al., 2011; Shiferaw et al., 2019). Informational entropy is defined as the average amount of information of a random variable. Entropy is higher when uncertainty of an outcome is high and thus when events carry relatively more information. Entropy is maximal for variables with a uniform distribution. In terms of visual exploration patterns, a higher spread or a broader spatial distribution of gazed locations results in higher entropy. Conversely, a narrow spatial distribution results in lower entropy values.

To calculate entropy values for each subject and image, first, a discrete spatial distribution of gazed locations was constructed by dividing each image into a 20 × 20 matrix of 2° x 2° cells. Next, we counted how many times each cell was gazed at by a given participant. Finally, the entropy value of each spatial distribution was calculated by the following:

$$H\left(P\right)=-\overset{n}{\underset{i=1}{\sum }}\frac{p_{i}^{\text{cs}}*\log_{2}(p_{i}^{\text{cs}})}{\text{Coverage}(p_{i}^{\text{cs}})},$$

where $p_{i}^{\text{cs}}$ is the probability of gazing at a given cell. Coverage is a correction term suggested by Chao and Shen (2003) as a modification of the original entropy formula to avoid biases because of limited sampling (Wilming et al., 2011).

To confirm the robustness of the results, entropy values were additionally calculated based on gaze distributions for a smaller (1° × 1°) and larger (4° × 4°) cell size. Results did not differ, and thus we report the results based on a cell size of 2° × 2°.

#### Predictor maps for visual exploration patterns

We evaluated how well each participant’s exploration patterns were explained by the following: (1) the exploration pattern of other participants; (2) the low-level features; and (3) the high-level visual features of the presented images. The following three different predictor maps were correspondingly generated: (1) the visual exploration pattern for each image as assessed in the SC control group; (2) the low-level Intensity Contrast Feature (ICF) model of the images (Kümmerer et al., 2017); and (3) the high-level feature map for the images, as defined by the DeepGaze II (DG-II) model (Kümmerer et al., 2016).

The first predictor was derived from the empirical distribution of gaze locations across all participants of the SC group. A two-dimensional spatial probability distribution was constructed for each image by pooling all the gaze eye-tracking samples of SC individuals for each image. For predictions within the SC group, the SC predictor map was constructed in a leave-one-out cross-validation procedure. Pixel-level gaze counts were spatially smoothed with a two-dimensional Gaussian unit kernel (full-width at half-maximum = 2°) and normalized by dividing by the total count of gaze samples.

The second predictor map, ICF, consisted of a two-dimensional spatial distribution constructed based on the low-level features of images (luminance contrast). Different low-level features are known to be highly correlated (Onat et al., 2014), and simple models based on contrast features seem to perform as well as more complex models that include multiple low-level visual features (Kienzle et al., 2009). Thus, we used a low-level model based solely on luminance contrast.

The third predictor map, DG-II, consisted of a two-dimensional spatial distribution constructed based on features derived by a deep neural network optimized for object recognition (Simonyan and Zisserman, 2014). The DeepGaze II model is currently considered the best performing model for free viewing according to the MIT Saliency Benchmark 2019 (http://saliency.mit.edu/). The DG-II model selects local features that serve as a basis for object classification, but it does not segment or tag objects. Note that the DG-II model typically performs the best at predicting eye movement behavior for images depicting text or faces, which our stimuli dataset did not include. Nevertheless, the DG-II model has been shown to outperform the ICF model even in the absence of such features (Kümmerer et al., 2017).

ICF and DG-II maps were computed for each image using the Python code made available by Matthias Kümmerer and the Bethge Laboratory (https://deepgaze.bethgelab.org). ICF and DG-II maps were generated for each original image, as well as for three low-pass-filtered image versions. The latter were obtained by filtering the images using a 2D Gaussian kernel with frequency cutoffs (reduction, 0.67) at 0.5, 1, and 2 visual degrees, respectively.

#### Area under the curve

To determine how well a given predictor map explained participants’ visual exploration patterns, we tested whether the values of the predictor map allowed a classification of image locations as gazed versus nongazed. For nongazed locations, values were taken from gazed locations in other images by the same participant. This procedure to define nongazed locations was introduced to avoid an inflated classification success because of possible spatial biases (Tatler et al., 2005; Wilming et al., 2011; Bylinskii et al., 2016), in both human visual exploration patterns and photographic image features (Tatler et al., 2005; Tatler, 2007; Einhäuser et al., 2008b). For each participant, gazed and nongazed values were pooled across images. These values were used to estimate the classification success of a predictor map by calculating the area under the curve (AUC) of the receiver operator characteristic curve (Green and Swets, 1988; Fawcett, 2006). AUC values can be calculated by first taking the Mann–Whitney *U* statistic (also called Wilcoxon rank-sum test) between gazed and nongazed values of the predictor map, as follows:

$$U=R_{\text{gazed}}-n_{\text{gazed}}(n_{\text{gazed}}+1)/2,$$

where $n_{\text{gazed}}$ is the sample size of gazed locations and $R_{\text{gazed}}$ is the sum of ranks in the sample of gazed location, obtained by assigning a numeric rank to every gazed and nongazed values, beginning with 1 for the smallest value. AUC values are directly derived from *U* by normalizing with the product of the number of gazed and nongazed locations (Bamber, 1975), as follows:

$$AUC=U/(n_{\text{gazed}}*n_{\text{non\textbackslash{}\_gazed}}).$$

AUC values range between 0 and 1, with 0.5 corresponding to chance discrimination and 1 indicating perfect classification. In some analyses, AUC values were obtained per image rather than per subject: gazed and nongazed values were pooled across participants for each image instead across images for each participant.

To further control for any additional potential analyzing bias, control AUC values were calculated as follows: instead of using the predictor map for a given image, images were shuffled; that is, the predictor map of another randomly selected image was used to predict visual exploration of a given image.

#### Statistical tests

Group differences in instantaneous gaze velocity, entropy, and AUC values were evaluated with robust linear regression models using a categorical group factor. The models used an iteratively reweighted least-squares method using a bisquare weight function, as implemented in the MATLAB R2019b function *fitlm* (Holland and Welsch, 1977). As there were six possible between-group comparisons, group contrasts were tested at a Bonferroni-corrected significance level of 0.05/6.

Moreover, AUC values for the SC, ICF and DG-II predictor maps were evaluated for different time periods after image presentation. This analysis tested whether classification success depended on the phase of visual exploration. AUC values were computed from data partitions obtained by dividing each participant’s gaze data into eight nonoverlapping 500 ms intervals, from the beginning to the end of the trial. These sets of AUC values, excluding the first interval, were entered in a linear mixed-effects model with group as a categorical factor, a time interval covariate as a fixed effect (seven levels), and participant identity as a random effect.

In the CC and NC groups, we additionally evaluated differences in AUC values generated from gaze locations, obtained by dividing each participant’s gaze data into 10 bins according to the magnitude of instantaneous gaze velocity. This analysis tested whether classification success of the SC predictor map depended on gaze stability in the CC and NC group. The new set of AUC values were entered in a linear mixed-effects model with group as a categorical factor, a velocity quantile covariate as a fixed effect, and participant identity as a random effect.

To assess the effects of short-term memory on visual exploration patterns, the repetition effect was evaluated by comparing the entropy values between the first and the second image of trial pairs. Entropy values for each image were entered in a linear mixed-effects model, with participant group (four levels: CC, SC, DC, NC) and image order within a pair (two levels: first or second) as fixed effect predictors, and participant identity as a random effect. This analysis was performed separately for each type of trial pair (repeated identity, repeated category, unrelated new image).

Linear mixed-effect models were calculated in R (version 3.6.3), using restricted maximum likelihood estimation as implemented in the *lme4* package (Bates et al., 2015). The reported *p*-values were based on the *t*-distribution using degrees of freedom calculated with the Satterthwaite method, as implemented by the *lmerTest* package (Kuznetsova et al., 2017).

Differences between groups in object recognition performance were evaluated with a generalized linear model using a binomial distribution and a logit link function, as implemented in the R *stats* package. The same procedure was used to evaluate the association between visual acuity and the AUC values in CC participants. Detailed specifications and output summaries of all models are described in the corresponding extended data figures.

### Data availability

The code for the statistical analyses, figures, and the anonymized, preprocessed data are available at the Research Data Repository of the University of Hamburg (doi: 10.25592/uhhfdm.1520). Original eye-tracking datasets are available on request from the corresponding author.

## Results

### Gaze stability is severely affected in CC participants

**Figure 1.**
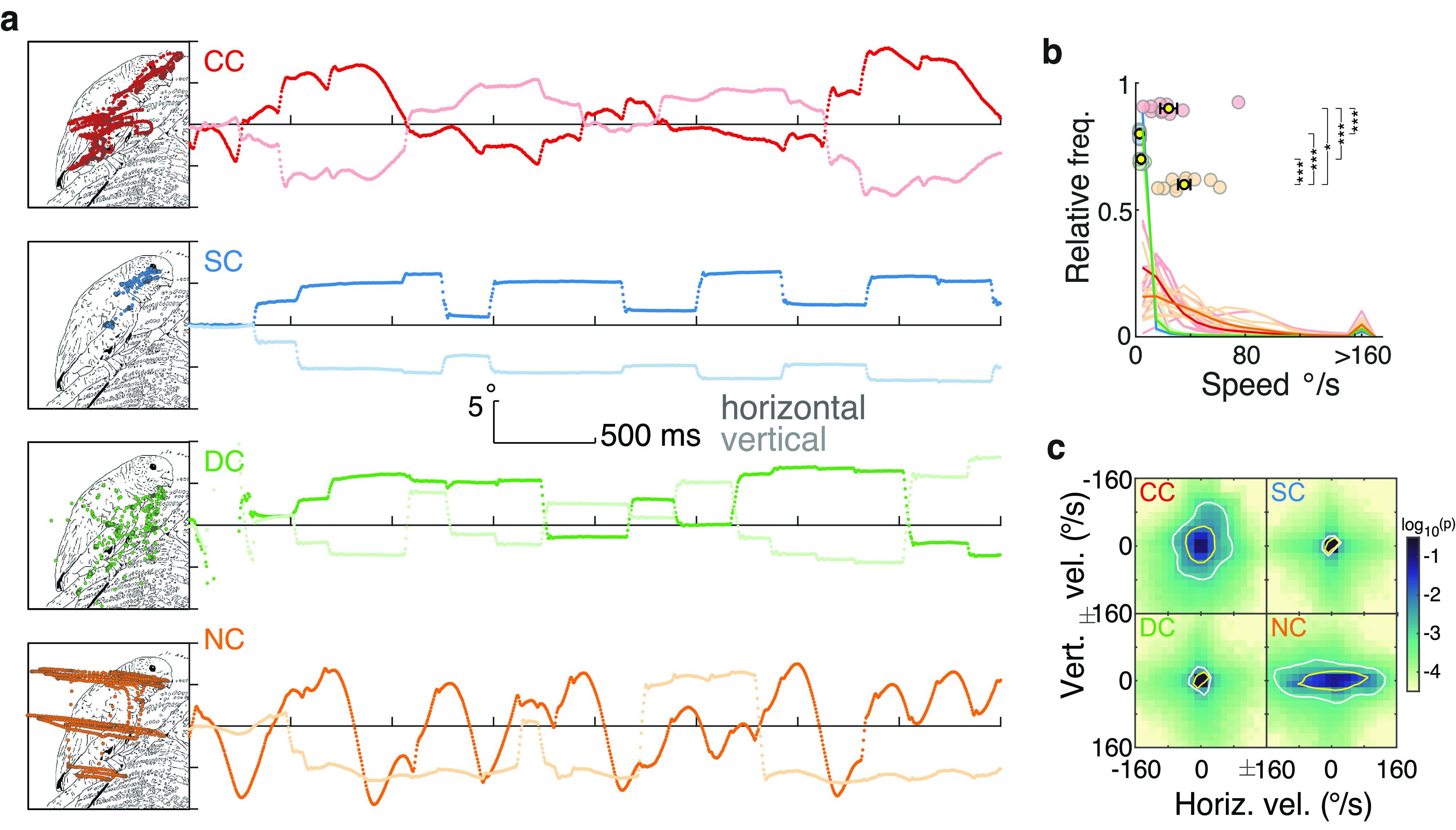
Eye movement kinematics during the visual exploration of an example image. ***a***, Examples of eye movement recordings of one participant from each group. Images were explored for 4 s. The left panels depict the gaze traces overlaid on a line-drawing sketch of the original photographic grayscale image; note that participants watched the original grayscale images. The right panels show eye movement traces as they progress over time and space along the horizontal (dark lines) and vertical (light lines) dimension. Extended Data Figures 1-1 and 1-2 show two other examples of eye movement recordings. ***b***, Distribution of the magnitude of instantaneous gaze velocity. Light lines indicate each participant’s distribution, and dark lines each group’s average distribution. Colored circles display each participant’s median value, and the yellow dots and error bars display the group’s mean and SEM (Extended Data Fig. 1-3, statistics). ***c***, Distribution of instantaneous gaze velocity (bin size, 16°/s; densities were individually generated for each participant and then averaged across the participants of each group). The color scale indicates the probability of a given gaze velocity in log_10_ scale. Yellow and white contours indicate areas that span ∼75% and 90% of the distribution. In all figures, significant contrasts among groups are indicated as follows: *p < 0.01, **p < 0.001, ***p < 0.0001, respectively.

As expected from the prevailing sensory nystagmus, eye movement trajectories were considerably altered in the CC group. Figure 1*a* displays examples of a single trial eye-tracking recording from one participant of each group (Movies 1, 2, Extended Data Figs. 1-1, 1-2). SC and DC participants showed the prototypical gaze kinematics of visual exploration of static images: their gaze movements were characterized by periods of high stability (i.e., fixations) interrupted by short periods of displacement at a high velocity (i.e., saccades). By contrast, CC and NC participants’ gaze movements were in a continuous, periodic displacement, as is typical of nystagmus. Participants’ gaze stability was quantified in terms of instantaneous gaze velocity; that is, how fast and in which direction the eyes moved from one moment to another (see subsections Data analysis and statistics, and Instantaneous gaze velocity). The magnitude of instantaneous gaze velocity was significantly higher in CC individuals compared with SC individuals (robust linear model contrast, *p *<* *0.001; Fig. 1*b*, Extended Data Fig. 1-3, full statistical results) and DC individuals (*p *<* *0.001), but was lower than in NC individuals (*p *=* *0.004). Gaze velocities showed no clear direction in CC individuals, whereas for NC individuals, gaze velocities were mostly along the horizontal direction (Fig. 1*c*). Such a pattern in NC individuals is typical of horizontal jerk nystagmus (Abadi and Bjerre, 2002).

10.1523/ENEURO.0051-22.2022.f1-1 Figure 1-1 Examples of eye movement recordings of one participant from each of the four groups. Download Figure 1-1, EPS file.

**Figure 2.**
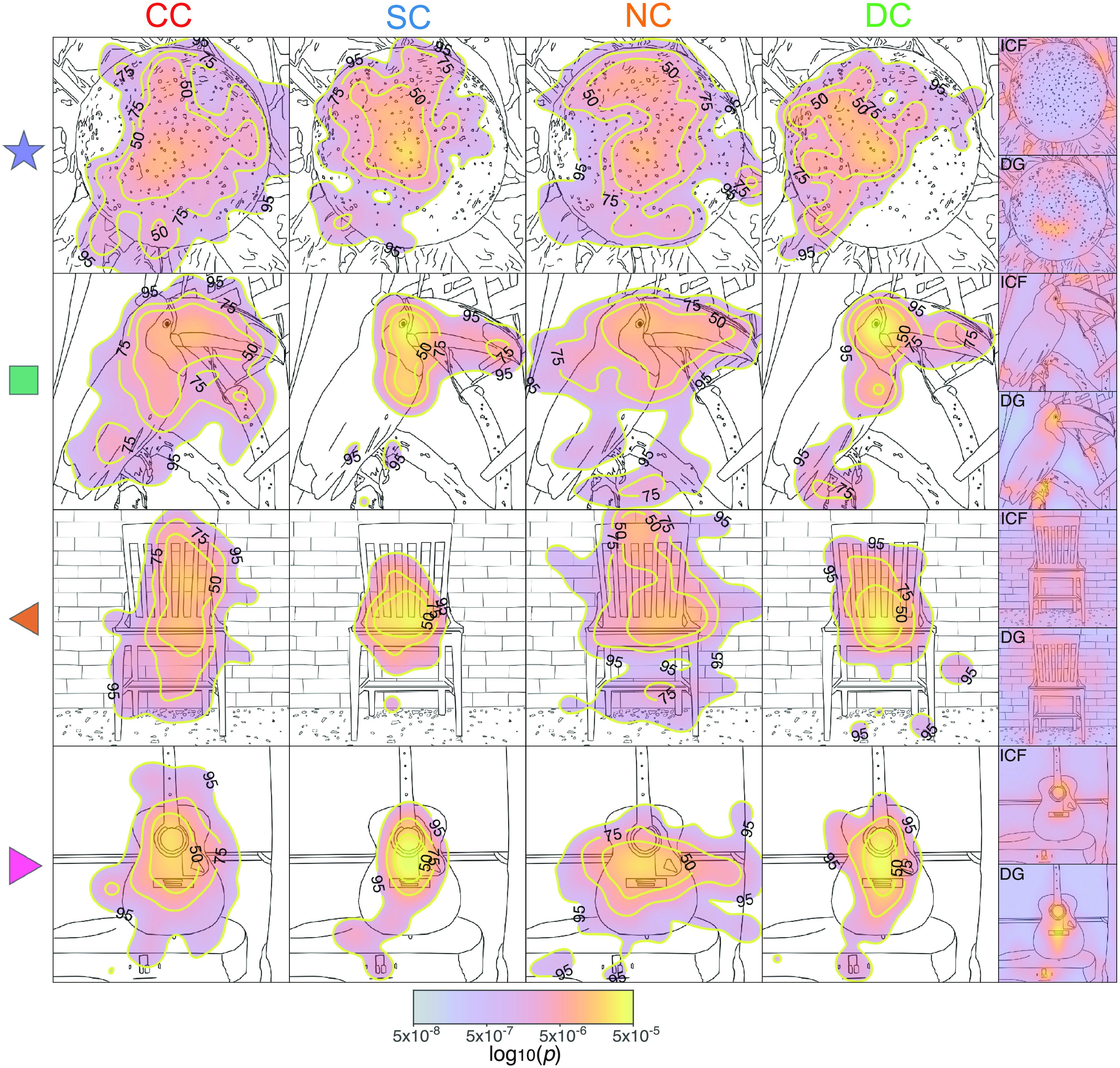
Examples of visual exploration by group. The subpanels show, for different images and the four groups of participants (Fig. 1, description), the spatial distributions of the probabilities to gaze different locations (pooled across participants and smoothed with a 2D Gaussian unit kernel), superimposed over line-drawing sketches of the original images. Warmer colors indicate a higher probability to gaze a location. Yellow contours indicate areas that span the top 50%, 75%, and 95% of the spatial distribution. As this distribution is constructed from all gaze eye-tracking samples (each occurring every 2 ms), these maps are equivalent to the spatial distributions of dwell time. The mean of entropy and AUC values for each of the four images are indicated by the corresponding symbol (star, square, and left and right pointing triangles) in Figure 3, *b* and *f*. The last column shows the DG-II and ICF predictor maps for each image. Extended Data Figure 2-1 shows the grand average of the spatial distributions of the probability to gaze a certain location across all images separately for each of the four groups. In addition, the corresponding grand average DG-II and ICF predictor maps are displayed.

10.1523/ENEURO.0051-22.2022.f1-2 Figure 1-2 Examples of eye movement recordings of one participant from each of the four groups. Download Figure 1-2, EPS file.

**Figure 3.**
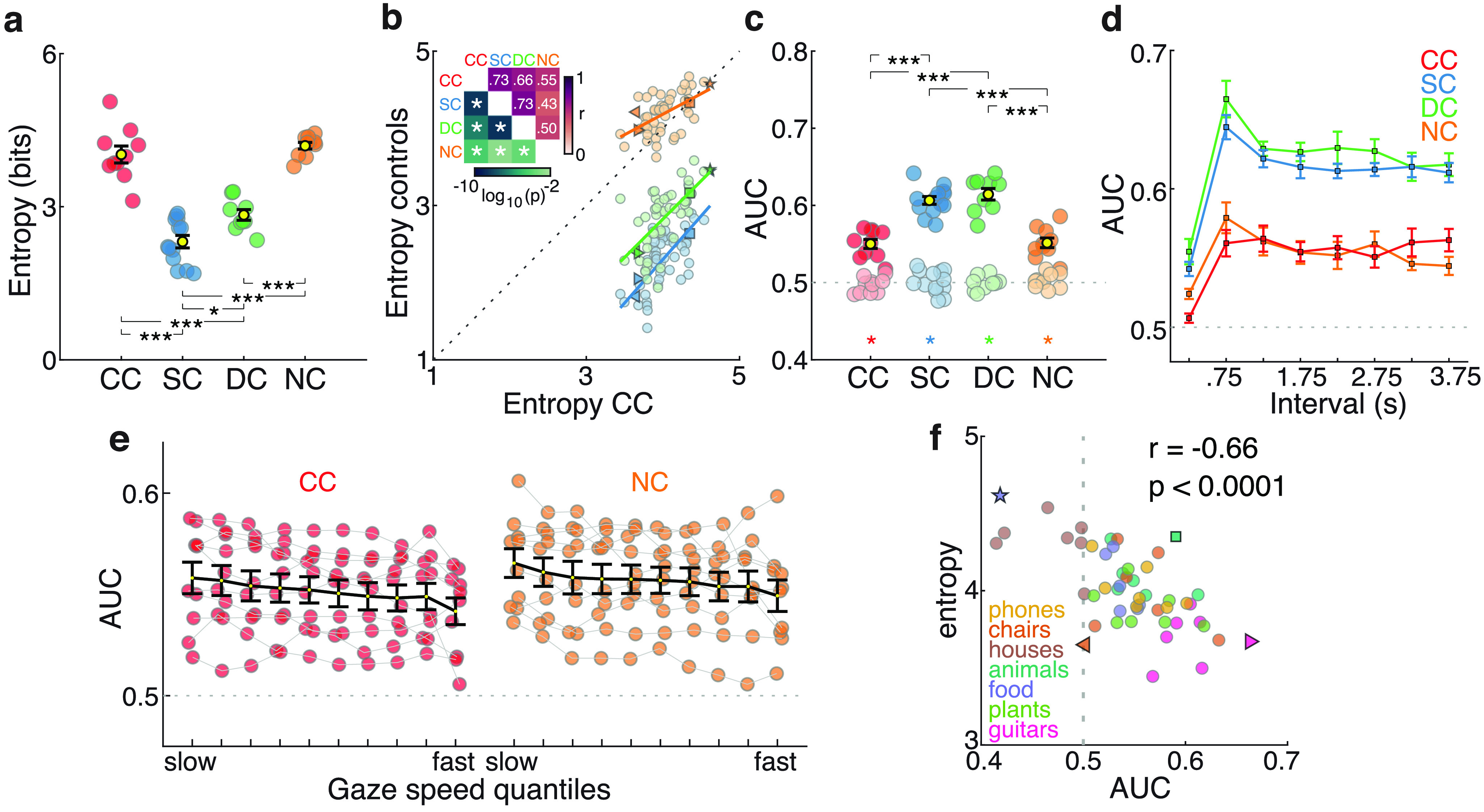
Spatial spread and predictability of visual exploration patterns. ***a***, Mean gaze entropy for each group (yellow dot with error bars, indicating the SEM) as well as for individual participants (colored dots; Extended Data Fig. 3-1, statistics). ***b***, CC participants gaze entropy per image compared with the gaze entropy values of the other three control groups. Colored continuous lines indicate a linear regression line for entropy values of the CC group (*x*-axis) and each one of the three control groups (SC, blue; DC, green; NC, orange). The top left inset depicts the corresponding Pearson’s correlation values (in a red scale, top right corner) and the corresponding *p*-values (in green, lower left corner). Asterisks indicate significant correlations after controlling for multiple comparisons (α = 0.05/6). ***c***, AUC values of the SC predictor map per participant and group. Dark-colored dots indicate AUC values for individual participants as derived by the predictor maps of the SC group to classify gaze and nongazed location. Light-colored circles from the corresponding AUC values for the control analysis in which image correspondence was shuffled. Bottom, Colored stars indicate that actual and control analysis values significantly differed. The control analysis values were not different from 0.5 (chance level). Extended Data Figure 3-2 shows statistics, and Extended Data Figure 3-3 shows the relationship between AUC values and different CC participants’ characteristics. ***d***, AUC values of the SC predictor map across time. Curves show, for each group, AUC values calculated from consecutive 500 ms data partitions (Extended Data Fig. 3-4, statistics). ***e***, AUC values of the SC predictor map as a function of instantaneous gaze velocity. SC predictor maps were used to calculate gaze in CC individuals separately for 10 quantiles of instantaneous gaze velocity (Extended Data Fig. 3-5, statistics). Extended Data Figure 3-6 shows the relationship between gaze velocity during fixations (SC and DC groups) and CC and NC participants’ first and second instantaneous gaze velocity quantiles. ***f***, Correlations of entropy and AUC values across all images for the CC group. Different object categories are color coded. Extended Data Figure 3-7 shows the same correlation for SC, NC, and DC groups.

10.1523/ENEURO.0051-22.2022.f1-3 Figure 1-3 Instantaneous gaze velocity statistical result. Download Figure 1-3, DOCX file.

Movie 1. Examples of visual exploration patterns. Each subpanel shows the exploration of one participant for the complete period of image presentation. Each red dot represents one eye-tracking gaze sample (downsampled from 500 to 125 Hz for better visualization) overlaid on a line-drawing sketch of the original photographic grayscale image; note that participants watched the original grayscale images. 10.1523/ENEURO.0051-22.2022.video.1

Movie 2. Examples of visual exploration patterns. Same as in Movie 1. 10.1523/ENEURO.0051-22.2022.video.2

In sum, these results confirm that, in contrast to SC and DC participants, the gazes of CC and NC participants were in a state of continuous motion; that is, gaze stability was reduced in these two groups.

### Visual exploration patterns of CC participants are stimulus driven and similar to those of control subjects

Four examples of group-pooled exploration patterns overlaid over line drawings of the original grayscale images are depicted in Figure 2. These images illustrate the resemblance of visual exploration patterns across groups. To quantitatively evaluate whether visual exploration patterns for natural scenes were stimulus driven in CC participants and to what degree they followed the same principles as in normally sighted control subjects, informational entropy was derived to parametrize the width of the spatial distribution of gazed locations (Shiferaw et al., 2019). Low entropy scores indicate a low degree of randomness of visual exploration patterns (see subections Data analysis and statistics, and Entropy).

10.1523/ENEURO.0051-22.2022.f2-1 Figure 2-1 Exploratory and feature bias. ***a***, Grand average, across all image and participants (per group), of the spatial distributions of the probability to gaze. ***b***, Grand average, across all images, of DG-II and ICF predictor maps. Download Figure 2-1, EPS file.

As expected from the nystagmus-related gaze instability, visual exploration by CC individuals covered a wider area of the images than visual exploration by SC individuals. CC participants’ entropy values were higher than those for SC and DC participants (robust linear model contrasts, both *p *<* *0.001; Fig. 3*a*, Extended Data Fig. 3-1, statistics), but were not different from those of the NC participants (*p *= 0.27). Thus, higher entropy values in the CC group were a consequence of nystagmus, rather than of congenital visual deprivation.

10.1523/ENEURO.0051-22.2022.f3-1 Figure 3-1 Entropy statistical result. Download Figure 3-1, DOCX file.

10.1523/ENEURO.0051-22.2022.f3-2 Figure 3-2 AUC (SC predictor map) statistical result. Download Figure 3-2, DOCX file.

10.1523/ENEURO.0051-22.2022.f3-3 Figure 3-3 ***a–d***, Relationship between SC predictor map AUC values in CC individuals and logMAR visual acuity (***a***), age at testing (***b***), age at surgery (***c***), and time from surgery (***d***). Download Figure 3-3, EPS file.

**Figure 4.**
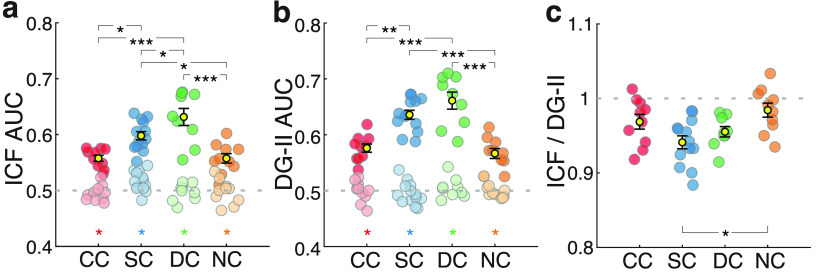
Degree of explained visual exploration behavior for low-level and high-level visual information and context. ***a***, AUC values resulting from the low-level ICF predictor maps (Extended Data Fig. 4-1, statistics). ***b***, AUC values resulting from the high-level DG-II predictor maps (Extended Data Fig. 4-2, statistics). ***c***, Ratio between ICF and DG-II AUC values (Extended Data Fig. 4-3, statistics). Extended Data Figures 4-4, 4-5, and 4-6 show AUC values of the ICF and DG-II predictor map across time and the corresponding statistics. Extended Data Figure 4-7 shows the ratio between ICG and DG-II AUC values obtained from low-pass-filtered versions of the images and the AUC values obtained from the nonfiltered images (Extended Data Figs. 4-8, 4-9, statistics).

10.1523/ENEURO.0051-22.2022.f3-4 Figure 3-4 AUC (SC predictor) per time interval statistical result. Download Figure 3-4, DOCX file.

**Figure 5:**
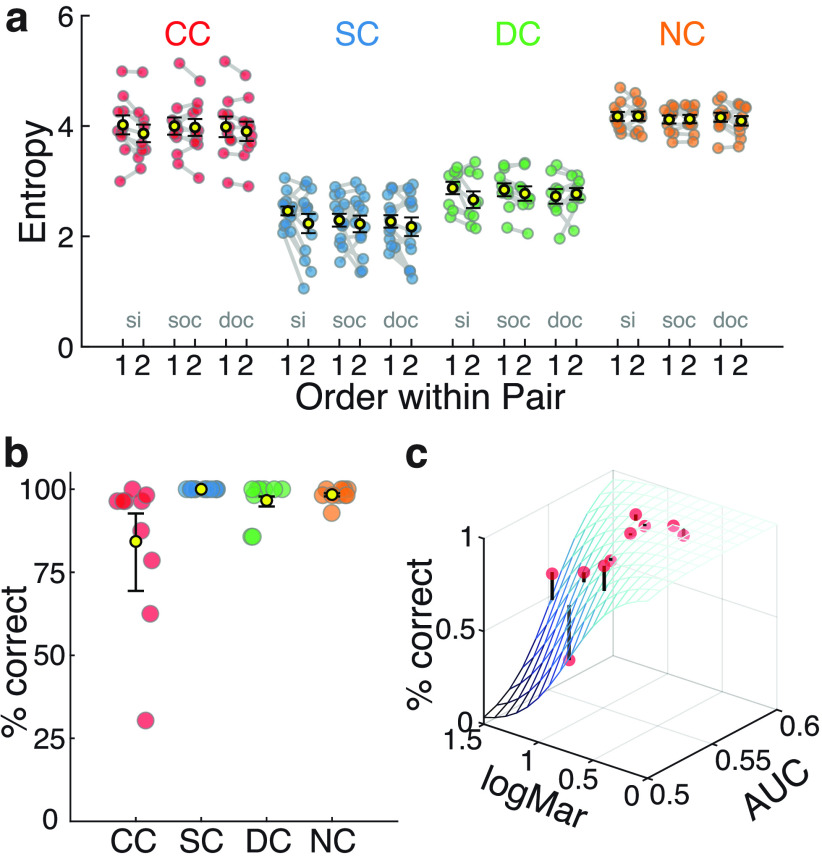
Effect of stimulus repetition and object recognition performance. ***a***, Gaze entropy for the first versus the second presentation of the same image (si), different images from the same object category (soc), and different images from different object categories (doc; Extended Data Fig. 5-1, 5-2, 5-3, statistics. ***b***, Percentage of correct images recognized for in each group (mean group performance in black with error bars indicating the SEM; Extended Data Fig. 5-4, statistics). ***c***, Recognition performance, visual acuity (logMar) and AUC values (obtained using SC predictor maps) for each CC individual. The blue shade mesh depicts the generalized logistic fit. Black lines starting at the red dots indicate the discrepancy between actual performance of a CC participant and model predictions (Extended Data Fig. 5-5, statistics). Extended Data Figures 5-6 and 5-7 show the relationship between performance and age at testing.

10.1523/ENEURO.0051-22.2022.f3-5 Figure 3-5 AUC (SC predictor) per velocity quantile statistical result. Download Figure 3-5, DOCX file.

10.1523/ENEURO.0051-22.2022.f3-6 Figure 3-6 Fixational gaze velocity SC and DC groups and first and second quantiles of gaze velocity in the CC and NC groups. Top, The grand average distribution, for the SC and DC groups, of the magnitude of instantaneous gaze velocity for samples corresponding to fixations. Bottom, Gaze instantaneous velocity first and second decile values for each CC and NC participant. Download Figure 3-6, EPS file.

10.1523/ENEURO.0051-22.2022.f3-7 Figure 3-7 Correlation across images between entropy values and AUC values. ***a–c***, SC (***a***), NC (***b***), and DC groups (***c***). Download Figure 3-7, EPS file.

Importantly, the entropy values of the CC group for individual images were significantly correlated with entropy values of the same images for the three control groups (Pearson’s *r*, all > 0.5, *p *<* *0.003; Fig. 3*b*). Thus, the relative extent of visual exploration of images was correlated across groups. This correlation suggests that visual exploration by CC individuals was strongly dependent on the characteristics of the images, and that this dependency was qualitatively similar to the dependency on image characteristics that guided visual exploration in control individuals.

Since stimulus entropy assesses the extent of visual exploration, but not the precise locations of gaze shifts, similar entropy values across groups do not unambiguously indicate the same visual exploration patterns. Therefore, we additionally evaluated whether the exploration patterns of the CC group were predicted by the corresponding visual exploration patterns of the SC group. For each image and participant, we used the pooled spatial distribution of gaze locations from the SC group to create an SC predictor map. We then used the latter to predict whether or not an image location was visually explored by CC individuals. Classification success was quantified by the AUC of the receiver operator characteristic (values >0.5 indicate correct prediction; see subsections Data analysis and statistics, and AUC; Swets, 1988; Fawcett, 2006).

SC predictor maps discriminated the gazed versus not-gazed locations above chance of the CC group (all groups AUC values, >0.5; one-sample *t* tests, *p *<* *0.001; Fig. 3*c*). To exclude the possibility that common image characteristics or spatial biases artificially enhanced prediction success, we ran a control analysis in which images were shuffled and AUC values from arbitrarily assigned images were derived. No successful prediction was achieved with these values (none of the AUC values in either group differed from 0.5, *p *>* *0.05). Although CC participants’ AUC values were overall lower than those for SC and DC participants (robust linear model contrasts, both contrasts, *p *<* *0.001; Extended Data Fig. 3-2, for statistics), they did not differ from those of the NC participants (*p *=* *0.93).

In the CC group, AUC values were not correlated with visual acuity (Pearson’s *r*_(8)_ = −0.19, *p *=* *0.59; Extended Data Fig. 3-3), age at testing (*r*_(8)_ = −0.05, *p *=* *0.87), age at cataract surgery (*r*_(8)_ = −0.28, *p *=* *0.42), or time since sight restoration (*r*_(8)_ = 0.2, *p *=* *0.56).

The previous analyses were based on the complete duration of a trial (4 s). To evaluate possible group differences in the temporal dynamics of visual exploration, we additionally ran the same analyses for the SC predictor maps separately for consecutive, nonoverlapping 500 ms time intervals. For the first interval (0–500 ms after image presentation), all groups had low AUC values (AUC values, ∼0.5; Fig. 3*d*). This result is consistent with previous findings and is most likely because of the starting position being forced to be at the center of the image and thus independent of image content (Schütt et al., 2019). After the first interval, CC participants’ AUC values increased, and remained at the same level throughout image presentation. By contrast, SC, DC, and NC participants reached their highest AUC values in the second interval (500–1000 ms), following which AUC values that progressively decreased until the end of image presentation. This group difference in the dynamics of visual exploration was confirmed by a mixed-effects model with a categorical predictor participant group, a time interval covariate (excluding the first interval), and participant identity as random effect: CC participants’ estimate of a time interval covariate was not different from 0 (*p *=* *0.9; Extended Data Fig. 3-4, statistics). In other words, there was no relationship between time interval and AUC values in the CC group. By contrast, all of the other groups showed a significantly more negative estimate of the time interval covariate than CC participants (all contrasts, *p *<* *0.006), indicating a decrease of AUC values as exploration progressed in the control groups.

Previous research suggested that visual acuity depends on the extent of the stable, “foveation,” period of the nystagmus (Dell’Osso and Daroff, 1975; Dell’Osso and Jacobs, 2002; Felius et al., 2011). Therefore, it is possible that CC and NC participants mainly explore the image during low-velocity periods of their nystagmus. To test this hypothesis in CC and NC participants, AUC values were separately calculated for 10 data partitions according to the magnitude of the gaze instantaneous velocity (Fig. 3*e*). AUC values were above chance for each velocity bin. A mixed-effects model with the categorical predictor group (run only with CC and NC groups), a velocity quantile covariate, and participant identity as random effect, revealed a significant effect of speed quantile (*p* = 0.002; Extended Data Fig. 3-5, statistics), without a significant main effect of group (*p *=* *0.6) and without a significant interaction of group and the velocity quantile covariate (*p *=* *0.83). Therefore, across both groups, slower gaze velocities resulted in higher AUC values. The first two gaze velocity quantiles of CC and NC individuals were approximately comparable to SC and DC individuals’ instantaneous gaze velocity during fixations (Extended Data Fig. 3-6). This result suggests that CC and NC individuals were able to systematically adjust visual exploration to gaze at the most relevant parts of an image during the low-speed phase of the nystagmus, when visual discrimination seemed to be best in individuals with nystagmus.

Entropy and AUC values were correlated in all groups, demonstrating that the lower the spread of visual exploration, the higher the agreement of visual exploration patterns across participants (Fig. 3*f*, results of the CC group, Extended Data Fig. 3-7, corresponding results of the other groups). This correlation did not differ between any of the four groups (comparison of Fischer’s *z*-transformed *r* values, all *p* > 0.05).

In summary, CC individuals gazed at similar locations of the image as normally sighted control subjects. These results support the hypothesis that the CC individuals’ visual exploration was based on the same underlying mechanisms. Thus, neither the acquisition of these representations, nor their use for visual exploration via eye movements, seem to require patterned vision at birth.

### Exploration patterns of CC participants are guided by both low-level and high-level visual features

Next, we evaluated to what degree visual exploration patterns were guided by low-level versus high-level visual information. For this purpose, predictor maps from two different saliency models were computed for each image: a first, low-level predictor map was constructed from local contrast as defined by the ICF model (Kümmerer et al., 2017). The second, high-level predictor map was constructed from features resulting from a deep neural network trained for object recognition, as defined by the DG-II model (Kümmerer et al., 2016, 2017; Schütt et al., 2019).

In all groups, visual exploration patterns were classified above chance by both the low-level ICF and high-level DG-II predictor maps (Fig. 4*a*,*b*; all AUC > 0.5, *p* < 0.001). The high-level DG-II model predicted the visual exploration patterns better (i.e., resulted in higher AUC values) than the low-level ICF model for all groups (paired *t* test: CC group, *p *=* *0.01; SC group, *p *<* *0.001; DC group, *p *<* *0.001) except for the NC group (*p *=* *0.13). We confirmed that this classification accuracy was not an artifact of general image characteristics or spatial biases: neither of the two models significantly predicted gaze patterns in either group after images were shuffled. These results suggest that CC individuals were able to make use of both low-level and high-level visual information for guiding visual exploration, similar to SC individuals.

10.1523/ENEURO.0051-22.2022.f4-1 Figure 4-1 AUC (ICF predictor map) statistical result. Download Figure 4-1, DOCX file.

10.1523/ENEURO.0051-22.2022.f4-2 Figure 4-2 AUC (DG-II predictor map) statistical result. Download Figure 4-2, DOCX file.

10.1523/ENEURO.0051-22.2022.f4-3 Figure 4-3 ICF/DG-II AUC ratio statistical result. Download Figure 4-3, DOCX file.

10.1523/ENEURO.0051-22.2022.f4-4 Figure 4-4 AUC values across time. Curves show, for each group, AUC values calculated from consecutive 500 ms data partitions. ***a***, ICF predictor. ***b***, DG-II predictor. Extended Data Figures 4-5 and 4-6 show statistics. Download Figure 4-4, EPS file.

10.1523/ENEURO.0051-22.2022.f4-5 Figure 4-5 AUC (ICF predictor) per time interval statistical result. Download Figure 4-5, DOCX file.

10.1523/ENEURO.0051-22.2022.f4-6 Figure 4-6 AUC (DG-II predictor) per time interval statistical result. Download Figure 4-6, DOCX file.

10.1523/ENEURO.0051-22.2022.f4-7 Figure 4-7 ***a***, ***b***, Ratio between AUC values for saliency predictor map obtained from low-pass-filtered versions of the images and the AUC values obtained from the nonfiltered images, for the ICF (***a***) and DG-II (***b***) predictor maps. ICF and DG-II saliency models were run with low-pass-filtered versions of the images using 0.5, 1, and 2 visual degrees spatial frequency cutoff. Group differences were evaluated separately per predictor map and filtered version with robust linear model contrasts. The ratio between ICF predictor map AUC values from images filtered at 0.5° and nonfiltered images was higher in the CC group compared with the SC group (*t*_(38)_ = –2.82, *p *=* *0.007; Extended Data Fig. 4-8, statistics) and the DC group (*t*_(38)_ = –3.51, *p *=* *0.001), but was not significantly different from the NC group (*t*_(38)_ = –1.06, *p *=* *0.29). No difference between groups was found for the 1° ratio or 2° ratio AUC. The ratio between DG-II predictor map AUC values from images filtered at 0.5° and nonfiltered images was higher in the CC and NC groups compared with the SC and DC group (all four contrast, *p *<* *0.007; Extended Data Fig. 4-9), but was not different in the comparison between CC and NC (*p *=* *0.79). The ratio between AUC values from images filtered at 1° and nonfiltered images was higher in the CC group compared with the DC group (*p *=* *0.005). No other group contrast was significant. No group difference was found for the DG-II 2°. In summary, compared with SC and DC participants, for CC and NC participants gazed and nongazed locations were to a larger degree predicted by the low spatial spectral content of the images. Download Figure 4-7, EPS file.

10.1523/ENEURO.0051-22.2022.f4-8 Figure 4-8 AUC (ICF predictor map low-pass filtered, 0.5) statistical result. Download Figure 4-8, DOCX file.

10.1523/ENEURO.0051-22.2022.f4-9 Figure 4-9 AUC (DG-II predictor map low pass 0.5 filtered, 0.5) statistical result. Download Figure 4-9, DOCX file.

AUC values were overall lower in the CC group compared with the SC and DC groups for both low-level and high-level predictor maps (robust linear model contrasts, all *p* < 0.004; Fig. 4*a*,*b*, Extended Data Figs. 4-1, 4-2, for statistics), while they did not significantly differ from the corresponding AUC values of the NC group (both *p* > 0.05). Importantly, the relative predictive power of the two models (ratio of the ICF and DG-II AUC values) was indistinguishable between the CC group and the three control groups (all *p* > 0.05; Fig. 4*c*, Extended Data Fig. 4-3, statistics). This confirmed that CC individuals weighted low-level and high-level visual information for guiding visual exploration similar to the SC, NC, and DC groups.

As in the analysis for the SC predictor map, we ran the analyses for the ICF and DG-II predictor maps separately for consecutive time intervals (Extended Data Fig. 4-4). For the low-level ICF predictor, the time interval covariate was not significant in any group (estimate not different from 0, all *p* > 0.1; Extended Data Fig. 4-5, statistics). For the high-level DG-II predictor, the SC group showed an effect of interval (*p *<* *0.001). By contrast, this effect was nonsignificant in the CC (*p *=* *0.29), DC (*p *=* *0.06), and NC (*p *=* *0.12) groups. Nevertheless, the estimate of the time interval covariate was more negative in the SC and DG groups than in the CC group (*p* < 0.0002 and *p* < 0.04, respectively; Extended Data Fig. 4-6, statistics).

Additionally, AUC values obtained from saliency predictor maps computed from low-pass-filtered images explained visual exploration in the CC and NC groups better than in the SC and DC groups (Extended Data Figs. 4-7, 4-8, 4-9, statistics). This is consistent with CC and NC individuals’ reduced visual acuity and reduced sensitivity to higher spatial frequencies (Ellemberg et al., 1999; Bedell, 2006; Hertle and Reese, 2007). Thus, it is justified to conclude that CC and NC individuals’ visual exploration predominantly made use of the low rather than the high spatial frequency components of visual stimulus features.

In sum, these results demonstrate a highly preserved ability of CC individuals to use both low-level and high-level visual information to guide visual exploration.

### Changes in visual exploration patterns for repeated images indicate visual short-term memory effects in CC individuals

Visual exploration patterns narrow down after an image has been repeatedly encountered (Noton and Stark, 1971; Ryan et al., 2000; Smith et al., 2006; Kaspar and König, 2011a,b). This result has been taken as evidence for visual exploration being guided not only by stimulus-driven factors but additionally by top-down factors. To assess such short-term memory effects, we analyzed differences in gaze entropy for two consecutive images, for which the second image was (1) identical to the first image, (2) a different image but displaying an item of the same category as the first image, or (3) a different, unrelated image.

Entropy values decreased between the first and second presentations of the same image in all groups, including the CC group (Fig. 5*a*). Furthermore, this reduction in the spread of visual exploration between repeated images did not differ between groups (no significant interaction between image repetition and group, *p* > 0.05; Extended Data Fig. 5-1, statistics). Importantly, in all groups, the reduction in entropy for consecutive images was specific for repeated images and did not generalize to category repetitions or different images (Fig. 5*a*, Extended Data Figs. 5-2, 5-3, statistics).

10.1523/ENEURO.0051-22.2022.f5-1 Figure 5-1 Entropy values for the first versus second image of a pair of identical images statistical result. Download Figure 5-1, DOCX file.

10.1523/ENEURO.0051-22.2022.f5-2 Figure 5-2 Entropy values for the first versus second image of a pair of images from same category statistical result. Download Figure 5-2, DOCX file.

10.1523/ENEURO.0051-22.2022.f5-3 Figure 5-3 Entropy values for the first versus second image of a pair of images from different category statistical result. Download Figure 5-3, DOCX file.

10.1523/ENEURO.0051-22.2022.f5-4 Figure 5-4 Performance for each group statistical result. Download Figure 5-4, DOCX file.

10.1523/ENEURO.0051-22.2022.f5-5 Figure 5-5 CC participants’ performance statistical result. Download Figure 5-5, DOCX file.

10.1523/ENEURO.0051-22.2022.f5-6 Figure 5-6 Performance versus age at testing. Age at testing was not correlated with performance across all participants (*r* = 0.06, *p* = 0.69) or when tested only for the CC group (*r* = 0.1, *p* = 0.78). For CC participants, a logistic regression model analysis showed no association between object recognition performance and age at testing (*p* = 0.17; Extended Data Fig. 5-7). Download Figure 5-6, EPS file.

10.1523/ENEURO.0051-22.2022.f5-7 Figure 5-7 CC participants’ performance and age statistical result. Download Figure 5-7, DOCX file.

In summary, CC individuals’ visual exploration patterns showed the same short-term memory-related reduction in spread as found in the control groups and demonstrated in previous research (Noton and Stark, 1971; Ryan et al., 2000; Smith et al., 2006; Kaspar and König, 2011a,b). This result suggests that CC individuals are able to integrate both stimulus-driven and top-down information from short-term memory to guide visual exploration.

### Object recognition performance is linked to systematic visual exploration in CC individuals

Object recognition performance was high in all groups (Fig. 5*b*). All SC participants, independent of their chronological age at testing, performed at 100%. Overall, the performance of the CC group (mean, 84.2% correct; range, 30.3–100% correct) was lower than in the three other groups (*p* < 0.001; Extended Data Fig. 5-4, statistics). For CC participants, a logistic regression model analysis revealed that object recognition performance was associated with better visual acuity (visual acuity predictor, *p* < 0.001; Fig. 5*c*, Extended Data Fig. 5-5, statistics) but not with age at testing (Extended Data Figs. 5-6, 5-7). Crucially, object recognition was additionally related to how well the CC individuals’ exploration patterns were predicted by the exploration patterns of SC participants (AUC predictor, *p* < 0.001). According to Akaike information criterion and Tjur *R*^2^ model-fit metrics (Extended Data Fig. 5-5), a model with both the visual acuity and AUC predictors performed better at explaining the object recognition scores of CC individuals than a model with either predictor in isolation. While high overall object recognition performance in CC individuals is in accordance with previous findings (Ostrovsky et al., 2009; Röder et al., 2013), this result further suggests that object recognition performance in CC individuals might benefit from systematic visual exploration.

## Discussion

Visual exploration of natural scenes by means of eye movements is guided by stimulus-driven mechanisms that make use of low-level and high-level visual features as well as by top-down mechanisms such as explicit goals and memory representations. The present study investigated the degree to which the development of the bottom-up and top-down mechanisms guiding systematic visual exploration of natural stimuli relies on early visual experience. Here, we tested visual exploration patterns in 10 individuals who had received delayed treatment for total dense bilateral congenital cataracts (CC group), some only in late childhood or adulthood. Participants watched close-up photographic images of different objects, plants, animals, and buildings. The visual exploration patterns of CC individuals were compared with those of a group of normally sighted control subjects (SC group), individuals treated for late-onset cataracts (DC group), and a group of individuals with pathologic nystagmus, but without a history of congenital cataracts or visual deprivation (NC group). We found remarkably preserved visual exploration behavior in the CC group, despite an absence of visual experience early in life. Indeed, CC individuals’ visual exploration patterns were successfully predicted by those of the SC group. The application of modeling approaches to identify the visual features guiding visual exploration revealed that CC individuals used both low-level and high-level visual information, and did so with a similar relative weighting as observed in the control groups. Furthermore, by analyzing the effects of short-term memory on visual exploration patterns, we demonstrated that CC individuals were able to integrate recently acquired memory representations with stimulus-driven visual information. Finally, despite the high object recognition scores of CC individuals, residual deficits were associated not only with their persistent lower visual acuity, but additionally were associated with the degree to which their visual exploration patterns resembled those of typically sighted individuals.

While most studies in sight-recovery individuals have focused on visual perceptual functions, the interaction of the visual and oculomotor system has hardly ever been investigated in this population. On one hand, this is surprising, given that visual perception crucially depends on overt exploration to align the gaze with the most relevant regions of the visual world. On the other hand, eye movements of sight-recovery individuals born with severe visual impairment or blindness are highly distorted because of a superimposing involuntary nystagmus, making them harder to assess (Abadi et al., 2006). The emergence of nystagmus in CC individuals is a direct consequence of visual deprivation within the first 8–12 weeks of life; the first 12 weeks of life are considered a sensitive period for the development of gaze stability control (Rogers et al., 1981; Gelbart et al., 1982; Lambert et al., 2006; Birch et al., 2009). We observed more irregular nystagmus in CC individuals than in NC individuals, whose nystagmus patterns of horizontal jerk movements with accelerating slow phases were characteristic of infantile nystagmus syndrome. While Abadi et al. (2006) did not directly demonstrate such irregularities in the nystagmus pattern of CC individuals, their study reported that, in accordance with our observations, more irregular nystagmus, that is, with multiplanar rather than uniplanar patterns, seems to emerge in severe cases of congenital cataracts.

To the best of our knowledge, the present study is the first demonstration that individuals with nystagmus, regardless of etiology, are able to systematically explore natural images despite nystagmus-related distortions. Previous research suggested that visual acuity in individuals with nystagmus depends on the duration of the “foveation” periods within their nystagmus (Dell’Osso and Daroff, 1975; Dell’Osso and Jacobs, 2002; Felius et al., 2011). In both the CC and NC groups, we observed that exploration was more predictable during low-velocity periods, that is during periods that, by and large, resemble foveation periods. Thus, individuals with nystagmus are capable of taking into account their idiosyncratic nystagmus pattern while exploring an image. However, it needs to be stressed that visual exploration was predictable in both the CC and the NC groups for the complete range of gaze velocities. This result is in agreement with more recent research on visual acuity during nystagmus, which indicated that visual perception is possible throughout the nystagmus cycle (Dunn et al., 2014).

While a qualitative assessment of simple ocular orienting to light is routinely performed in CC individuals during clinical examination, the presence of nystagmus has made it difficult to quantitatively study systematic eye movement behavior in this group. It was only recently that visually guided behavior was successfully assessed with eye tracking in CC individuals (Zerr et al., 2020). In this study, participants followed a salient, single visual target, which abruptly but regularly changed location. CC individuals showed intact visually guided eye movements, which were as precise and as fast as can be expected after taking their nystagmus into account. While such visually guided eye movements are likely a prerequisite for the exploration of natural scenes, they might be accounted for, to a large degree, by a simple reflexive mechanism based on luminance contrast. By contrast, real-world visual exploration is not just driven by low-level information such as luminance contrast, but additionally uses high-level features, and integrates top-down influences such as goals and prior knowledge retrieved from memory (Tatler et al., 2011; König et al., 2016; Veale et al., 2017). Since previous research has documented better recovery of low-level than high-level visual processing in CC individuals (McKyton et al., 2015; Sourav et al., 2020; Pitchaimuthu et al., 2021), we expected that visual exploration of natural images would be mostly guided by low-level visual features. Contrary to this hypothesis, CC individuals relied on high-level information, and used both low-level and high-level information in a manner similar to that of SC and DC control groups.

For all three predictor maps (SC group predictor maps, ICF and DG-II predictors), the AUC values were significantly higher than chance in predicting the gaze patterns of CC individuals. However, they were overall lower than what has often been reported in similar studies (Wilming et al., 2011; Bylinskii et al., 2016; Kümmerer et al., 2017). This might be because of the characteristics of the images and constraints of the present study. First, all images featured a single central object, which might have reinforced a visual exploration bias toward the center. Since our analysis procedure controlled for this potential central bias, it might have lowered AUC values in the present study. Second, grayscale images were presented, which attenuated features that strongly guide typical visual exploration (Onat et al., 2014). Third, our analysis was not based only on fixations, but rather considered all eye-tracking gaze samples of the complete trial, including saccades. This was necessary because of the prevailing nystagmus in the CC and NC groups, and for a uniformity of the analysis across groups. By contrast, almost all previous studies that evaluate free-viewing behavior are based on fixations excluding saccades, and often excluding the first fixation following image presentation.

Although we found overall broader and less well predicted visual exploration patterns in the CC group than in the SC and DC groups, CC participants’ visual exploration was overall comparable to visual exploration in the NC group. A difference between the CC and NC groups was, however, detected in a time interval resolved analysis: whereas in SC, DC, and NC participants exploration was more predictable at the beginning of visual exploration (500–1000 ms interval) than during later phases, CC participants showed consistent AUC values throughout the exploration period. Decreasing predictability of visual exploration has been observed in previous research (Onat et al., 2014; Schütt et al., 2019). This has been interpreted as an initial bottom-up orienting response, followed by a gradual broadening of visual exploration (Schütt et al., 2019). The initial strong bottom-up response has been shown to be a consequence of the use of high-level features, rather than primary low-level features (Onat et al., 2014; Schütt et al., 2019). Indeed, this is the pattern of visual exploration that was observed in the SC group. In contrast to the SC group, prediction accuracy driven by high-level features did not vary with time in the CC individual. Thus, we speculate that despite using high-level features for visual guidance, high-level information did not interact with the initial phase of bottom-up exploration in the CC group. Future research might confirm this observation, since the dynamic change of predictability of the high-level model was not significant in the DC and NC groups.

It is unclear to what degree CC individuals are capable of visually exploring more complex scenes (e.g., images with multiple items or images that are generally harder to perceive for them). In fact, as a recent study has reported that CC individuals conduct fewer eye movements to the eyes region (Zohary et al., 2022). In the present study, we also avoided high stimulus eccentricities because of well known deficits in the peripheral vision of CC individuals (Lewis and Maurer, 2005), which is likely enhanced by the effects of nystagmus (Chung and Bedell, 1995; Pascal and Abadi, 1995).

Stimulus-driven guidance of visual exploration is thought to emerge from topographical “feature maps” representing visual features such as color, orientation, luminance, and motion (Itti and Koch, 2001; Veale et al., 2017). It is assumed that these feature maps serve as a source for “saliency maps.” Saliency maps represent how conspicuous or “salient” different regions of the visual field might be (Koch and Ullman, 1985; Itti and Koch, 2000). Our results indicated that the emergence of both of these mechanisms—the extraction of visual feature maps as well as the computation of saliency maps—do not seem to depend on early visual experience during a sensitive period.

The computation of feature and saliency maps has been proposed to be followed by the derivation of a “priority map” (Bisley and Goldberg, 2010). Priority maps are thought to combine bottom-up stimulus-driven information and top-down constraints to select the next gaze location (Bisley and Goldberg, 2010; Veale et al., 2017). Top-down influences have often been studied by manipulating task instructions. A special case of a nonreflexive, implicit top-down influence on visual exploration is the effect of short-term memory: If an image is repeated, the distribution of gazed locations narrows down (Hannula, 2010). Short-term memory effects on visual exploration because of image repetition have been reported to be unrelated to changes in low-level visual features (Kaspar and König, 2011b), suggesting that these effects are neither because of low-level adaptation, nor because of a reweighting of low-level and high-level image features. Whether or not the CC group would show memory-based gains on visual exploration over longer delay periods, as used in previous studies (Hannula, 2010), or other task-based top-down effects might be investigated in future studies.

CC participants were able to recognize the visual stimuli, which is in agreement with previous reports showing that even after a long period of congenital blindness, sight-recovery individuals were able to correctly name everyday objects (Maurer et al., 2005; Ostrovsky et al., 2009; Röder et al., 2013) and to recognize artificial objects through temporal integration (Orlov et al., 2021). In contrast to normally sighted participants who performed at ceiling, the performance of the CC group on object recognition was not perfect in the present study. Crucially, better image recognition in CC individuals was associated not only with better visual acuity, but additionally with how much CC participants’ gaze patterns resembled those of normally sighted control subjects. Although this association must be considered preliminary because of the limited sample size in the present study, this finding is compatible with previous research. For ambiguous or noisy stimuli, visual exploration of diagnostic features precedes explicit recognition, rather than object recognition guiding exploration (Holm et al., 2008; Kietzmann et al., 2011). However, similar latencies of the N170 wave of event-related potentials, an electrophysiological component that has been associated with the structural encoding of objects, speaks in favor for a recovery of typical object recognition times in CC individuals (Röder et al., 2013). Since the overall low visual acuity in CC individuals can be considered analogous to noise, we speculate that visual exploration aided rather than interfered with object recognition in CC individuals.

The idea that visual exploration promotes object recognition is reminiscent of theories from developmental psychology on how infants learn to recognize objects. For example, information-processing accounts assume that object recognition emerges in an active interaction with the visual world (Johnson and Johnson, 2000; Johnson, 2001; Johnson et al., 2008). Object recognition advances with an improvement in active sampling, that is, in visual exploration. It has been hypothesized that newborns’ preference for edges and motion, as well as their ability for figure–ground segregation, acts as an initial guide for where to look (Slater et al., 1990; Johnson and Johnson, 2000; Johnson, 2001). Further, it was proposed that object-defining higher-level features are acquired while continuously exploring the visual world (Johnson and Johnson, 2000). For example, the level of object knowledge in 2- to 3.5-month-old infants (Johnson et al., 2004) and the ability to process facial expressions in 6- to 11-month-old infants (Amso et al., 2010) were found to depend on visual exploration patterns. Our results are consistent with the idea of active visual exploration being instrumental for the acquisition of object knowledge. We speculate that CC individuals’ postsurgery visual exploration might initially have made use of the same preferences for edges and motion as suggested for newborns (Johnson and Johnson, 2000; Johnson, 2001). This additionally requires functioning oculomotor control in CC individuals capable of taking nystagmus-related trajectories into account. As children refine visual exploration to rely more on high-level features (Açık et al., 2010; Helo et al., 2014), we assume the same for CC individuals following cataract removal surgery. Indeed, CC individuals of the present study who had acquired the most typical visual exploration patterns were those who performed the best at object recognition.

None of the measures tested (i.e., entropy, AUC, and performance) showed an association with age at testing or time since surgery in CC participants. At first glance, this result seems surprising given the large range of ages at testing and of time passed since surgery. However, the lack of such a significant association requires replication, since our sample size was limited by the availability of a rare population. Further, all CC participants were >10 years of age. Previous research has reported adult-like visual exploration in terms of entropy and AUC measures in children >7 years of age (Açık et al., 2010; Helo et al., 2014). Finally, we tested CC individuals at least 7 months postsurgery. Thus, the duration since surgery within which visual input was available might have been sufficient to acquire visual exploration strategies, and the associated object knowledge. In fact, previous research in cataract-reversal individuals who underwent a long period of visual deprivation has provided evidence that knowledge of object shape emerges within this time period (Wright et al., 1992; Ostrovsky et al., 2009; Held et al., 2011; Chen et al., 2016).

In conclusion, the remarkably preserved exploration patterns of sight-recovery individuals with a history of a transient phase of congenital patterned visual deprivation suggests that the development of visual exploration mechanisms does not depend on experience within a sensitive period. In contrast to prevailing deficits in visual acuity, gaze stability, and other high-level visual functions (Röder and Kekunnaya, 2021), visual exploration mechanisms seem to emerge after sight restoration. We speculate that similar to infants, the newly available, low spatial frequency information might initiate recovery in individuals with reversed congenital cataract; followed by refinement, as in typical ontogenetic development. Finally, it might be hypothesized that visual exploration after sight-restoration surgery might stimulate the acquisition of visual object knowledge despite visual acuity deficits and nystagmus.

## Synthesis

Reviewing Editor: Ifat Levy, Yale University School of Medicine Decisions are customarily a result of the Reviewing Editor and the peer reviewers coming together and discussing their recommendations until a consensus is reached. When revisions are invited, a fact-based synthesis statement explaining their decision and outlining what is needed to prepare a revision will be listed below. The following reviewer(s) agreed to reveal their identity: Ehud Zohary, John Greenwood.

The authors examine the eye-movement strategies used in the visual exploration of images by individuals born with dense cataracts who subsequently regained their sight. Though the visual abilities of similar individuals has been examined previously, their active exploration and eye-movement patterns were previously unknown. Since active vision is such a critical component of vision, it is a central question in sensory system development.

The cataract-recovery (CC) group are compared with a set of interesting control groups - sighted controls (SC), those with milder developmental cataracts that did not obscure sight (DC) and those with nystagmus (NC; uncontrolled eye movements of a nature similar to those in the CC group after cataract removal). The later nystagmus group are a particularly interesting inclusion given the many similarities in their active exploration with the CC group. The authors examine the exploration patterns not only through comparison between the gaze patterns of the CC and SC groups, but also with comparison to feature models of luminance contrast and higher-level saliency maps through the DeepGaze model. Their results suggest that these individuals have near-normal eye movements when freely exploring real-world images despite pervasive gaze instability. Specifically, their eye movement patterns were well accounted by predictors based on normally sighted controls and models based on low- and high-level visual features. Recent memory information also affected their scanning patterns as was the case in controls. The authors conclude that the development of efficient visual exploration patterns is not restricted by a limiting critical (or sensitive) period.

This paper studies a very unique population, allowing for studying issues in visual development that are normally intractable. The study’s main question is intriguing, and important, the analyses are creative and technically demanding, and the results are overall quite convincing. The authors have done a very thorough job using sophisticated data analysis tools, and the proper statistical techniques. They also address the issue of the image blur that the sight recovery patients suffer from, even after surgical treatment (though to a lesser extent than prior to surgery).

Reviewers have, however, identified several drawbacks, and noted the unclear description of the methodology that hindered adequate evaluation of the results and their interpretation, as detailed below.

1. Data presentation

The basic data on which the various analyses were done should be presented clearly. Figure 2E is important and should be presented and explained carefully in detail. It seems that the colors refer to the percentile of the specific pixel in the image (meaning that this is a relative measure). What about the absolute value (in terms of dwell time). This may differ significantly across groups and is currently not shown.

2. The similarity of exploration patterns between groups

The examination of eye movement parameters (as plotted in Figure 1) reveals differences between the groups, with continuous motion and greater entropy/spread in the CC and NC groups relative to the Sighted Controls, and an interesting difference between the properties of the nystagmus in CC (equivalent in all directions) and NC individuals (predominantly horizontal). However, the authors assert at several points that the visual exploration of the CC individuals is “indistinguishable from those of controls” (e.g. line 382). The measures of entropy indeed correlate between groups (p19) and the predictor map of the Sighted Controls can account for gaze positions at an above-chance level (p20), but this is not the same as the exploration patterns being indistinguishable. They are clearly similar, but there are also differences that are not being accounted for by the current measures (e.g. AUC values fall below in all cases).

This can be seen in Fig1A (& in the movie), which are useful for appreciating the raw data. Clearly, the SC participant focuses almost solely on the parrot’s eyes; The CC participant shows partial overlap but their gaze drifts to other parts of the animal. The DC participant shows no correspondence at all (very peculiar), & the NC case is dominated by the horizontal nystagmus. Maybe this is a non-classical example or choice of participants. A few more examples may help here.

One thing that comes to mind here is that it seems that the temporal order of the eye-movement behaviors is not considered in the analysis. Is it possible for instance that the CC individuals may scan the images in a different order than sighted controls, or that they may jump between locations more rapidly as they try to make sense of the scene, similar to the way that dyslexic readers make ‘re-fixations’ or ‘regressive eye movements’ during reading (Pavlidis, 1981)? It seems that the current analyses would be insensitive to these patterns given their dependence on spatial maps rather than temporal order.

Regardless, greater care should be taken in the statements made about the similarity between these groups. Greater discussion of the limitations of the current measures would also be ideal.

3. Limitations of the image set

Relatedly, the image sets here are quite simple and uniform in nature - all had a single object located near the center of the image (line 161). Moreover, the stimuli were presented for 4 sec - this is a very long time for recognizing a single object. It is likely that active vision contributes to object recognition only in the first few saccades in the first 1-2 sec. It is possible that CC participants require more time for object recognition than the controls. Controls may have accomplished the task quickly, and then directed their vision to other features, which were not part of the figure object. This could have bloated the exploration, matching it more closely to that seen in the CC group. Consistent with that, the AUC is quite low (< ∼0.65) even in the SC group, suggesting that there is quite a bit of variation in the scanning patterns between SC participants. It may be worthwhile to compare performance across various time windows to test this (say comparing eye movements in the first 2 sec, vs the last 2 sec).

Another helpful approach may be to “clean” the CC & NC data by deleting all periods in which the nystagmus was present, and focus on the samples in which there was minimal movement (using a criterion similar to the offline calibration technique, or using only time points in which the velocity was within the yellow contours of the velocity map of SC & DC). If the authors are correct, after such a correction, eye movements of these two groups (CC & NC) should be more closely matched to that of the controls. Indeed, the gaze speed quantiles analysis (Ext fig 2-1) points in that direction, but it is unclear if the slowest quantile is within the speed range of the other groups (SC, & DC). The authors used a similar approach to account for the fact that the participants from the different groups have different proportions of missing data, with the ones suffering from nystagmus (CC,NC) having much greater signal loss ∼10% than the other groups (DC,SC: ∼4%). Thus, in the CC &NC groups, the actual proportion of time spent on the objects (from the total stimulus presentation time of 4 sec) was less than reported in the paper, because the times in which gaze was outside the measuring window were discarded. This should be acknowledged.

In a similar vein, would the CC individuals show the same similarities to controls in more complex scenes? This would surely increase the complexity of the analyses, and the variability of associated eye movements, but individuals have nonetheless been found to show reliable individual differences in their exploration in more complex image sets (De Haas, Iakovidis, Schwarzkopf, & Gegenfurtner, 2019), e.g. with some being biased more towards faces and others to text, etc. Given that individuals themselves start to vary as images become more complex, is the similarity observed here simply an artefact of the limited stimulus type?

Similarly, would the CC individuals be expected to behave the same way in more complex images with a high degree of clutter? Given that visual crowding effects are elevated in individuals with nystagmus (Chung & Bedell, 1995; Pascal & Abadi, 1995) this may yield dissociations from typical adults when they struggle to make sense of more complex scenes. Can this possibility be excluded given the current image set?

As above, greater consideration of the limitations of the study would be ideal. These may not be easy questions to address based on the current dataset, but some consideration of issues along these lines and/or acknowledgement of the simple, uncluttered, and uniform nature of the image set would help to more appropriately place the results in the context of the broader literature. The discussion makes speculations on a range of issues including development and sight recovery without really considering the nature of the representations that are meant to be similar between these groups, nor the limitations of this conclusion based on the current analyses. More critical evaluation of the results would be ideal.

4. Analyses using the predictor maps

The calculation of the predictor maps using the Sighted-Control (SC) data, and the low- vs high-level feature models is clearly explained. It is not clear, however, how the data were compared with this. It would be helpful to have an example of the ICF & DG-II model expectations for one specific image, to allow intuitive comparison of the actual pattern of eye movements of each group (as shown in Fig 2e) to the two models. Currently, it is unclear how exactly the predictors from the two maps look like, and especially, what the clear differences between the two models are and where they stem from. Line 293 refers to the position “currently being tested” - does this mean that eye positions were evaluated against the predictor maps one at a time? What was the sampling rate of the recordings in this case - were they down-sampled temporally (as the example video appears to be) or was this performed at the 500Hz rate of the eye tracker? Relatedly, it is not clear how the false alarms and hits were determined (lines 295-296). If a single position at each time is being examined, is the hit rate simply the value of the current kernel/grid point, with all others set to 0, and the false alarms the opposite? Finally, is the percentage of these values taken across all time points within the presentation period, or is it averaged across an image before being entered into this analysis? The authors also compare the AUC to the shuffled case, in which the predictors were based on a different, randomly selected image from the image set. The objects, however, were located close to the center, and thus the objects in different images overlapped considerably. Shouldn’t we expect to see (on average) an AUC larger than 0.5 for the shuffled cases as well? Can the authors explain why this is not the case? This needs to be clarified (e.g. maybe show a heatmap of the “figure” overlap across all images).

5. Interpretation of the results

The authors argue that the higher AUC values for the high-level DeepGaze model shows that CC individuals ‘relied more on high-level visual features’ (lines 489 and 628). It is not clear that this is accurate. The DeepGaze model certainly shows impressive contextual modulations and other properties that mean it is not merely responding to low-level featural contrast, but it would also be sensitive to low-level featural contrast in some cases. It is true that the success of this model as a predictor shows that both low and high level features need to be considered in order to predict eye movement patterns, but that is not the same as saying that the observers rely more on high- than low-level features.

The authors assume that the visual exploration strategies have evolved after surgery. But this cannot be stated decisively - the patients were not completely blind before surgery. It is very possible that even in the presence of minimal perception (before surgery), oculomotor behavior was utilized to optimize vision (however limited this vision was). Indeed, as the authors state, the pre-operated patients do track light with smooth pursuit patterns (and some can count fingers from nearby). Also, if exploration strategies only evolved after surgery, one might expect that the AUC (& entropy) values would be positively correlated with the time from surgery. This is not the case (although clearly the very limited sample size makes it difficult to judge this; also please note that the data in extended Fig 2.2 are missing). The statement that (Line 701) “the remarkably preserved exploration patterns of sight-recovery individuals with a history of a transient phase of congenital patterned visual deprivation suggests that the development of visual exploration mechanisms based on low- and high-level visual features, as well as on short-term memory, do not require visual input during the initial phase following birth” is overly stated. These patients have some visual input (albeit very limited) prior to surgery. The authors ought to acknowledge this possibility in the discussion.

6. Calculation of ‘instantaneous gaze velocity’

The authors calculate the ’instantaneous gaze velocity’ of the eye in order to assess its movement speed at any one point in time. It is not clear exactly how the equation provided gives an estimate of speed, however. The authors report (line 226-227) that “the weighted sum of the position (in screen pixels) of 6 non-consecutive gaze samples” was taken. This would certainly give an estimate of gaze instability/variability, but as reported would not give velocity. Imagine two readings. One proceeds across 6 pixels consecutively: 1 2 3 4 5 6. The other jumps in steps of ∼2 pixels before reversing: 1 3 5 6 4 2. The 6 positions are the same and would yield the same result when put into the weighted sum, but the differences between them (and thus the speed) are almost twice as large in the second example. The only way this may work is if these were not positions but rather differences/offsets from the current eye position - then the values would both have a sign (needed to give direction, as reported) and incorporate the size of the jumps between timepoints. Is that what was done? If so, the equation should be clarified on p11. If not, then this measure should not be referred to as velocity, but rather instability/variability.

Relatedly, why were the positions/differences taken at n{plus minus}2, {plus minus}3, and {plus minus}4, excluding n{plus minus}1?

7. Eye-tracking calibration with nystagmus

It is extremely difficult to calibrate eye trackers with individuals whose eye movements are unstable, as for the current CC and NC groups, so this is well performed. Are these procedures similar to those reported recently for the calibration of individuals with nystagmus (Dunn et al., 2019; Rosengren, Nyström, Hammar, & Stridh, 2020)? Additionally, it is a nice touch to report the calibration error (line 184), but how was this quantified? That seems to require some ‘ground truth’ for where the subject is looking in order to be properly quantified?

8. Memory effects

The authors used a design in which in 25% of the trial pairs, the same image was repeated. They show that the entropy in the image repeat is smaller than in the first presentation. However, the two images are separated by a mere 1 sec, so making a strong case about memory from this data is somewhat misleading. Also, there is no reference in the results to the other (same category, different category) conditions. What was the reason for having them (priming effect? probably resulting in faster recognition times). A better approach might have been to show all the stimuli twice, with an intervening (fixed or variable) number of stimuli between them.

Minor comments

- With the nystagmus (NC) individuals, what form of infantile nystagmus syndrome (INS) did they have? INS can be associated with albinism, low vision, retinal dystrophies, or can be idiopathic (of no known cause). Which of these were associated with the individuals tested here? Given that the results of this group are so interesting it would be useful to be able to compare them more readily (and e.g. reported in Table 1) to the wider literature on individuals with INS.

- The DC group (with early cataracts of lower severity) appear more like controls than the CC group (with dense bilateral cataracts), which is attributed to the DC group having had some vision in early life. Their acuity is also better than the CC group, however. Given that acuity can predict performance in the CC group, can we be sure the group difference is due to the early history and not simply their acuity?

- “As all CC and NC individuals suffered from nystagmus, dependent variables were defined with respect to gaze rather than fixation and saccades.” It is not clear what exactly was done. What do you mean by gaze? Did the authors use the individual sample data (@500Hz)?

- “We compared the scalar value of instantaneous velocity (i.e., the magnitude of the vector composed by the horizontal and vertical components) and the overall distribution of the velocity vectors (combining magnitude and direction) between groups”. The second sentence is unclear. Velocity includes speed (magnitude) and direction (angle). What does the distribution refer to? You can have a polar plot in which both are depicted (as a probability map; similar to Fig 1c), is that what the authors mean? If so, please refer to it in the text to aid understanding.

- Consider presenting an example (Figure 2E) of the raw data before showing the data analysis results (Fig 2a-d; i.e. reverse the order of presentation). Also, it is unclear if the visual exploration patterns (in 2E) are the group average, or an example from an individual subject. Do the colors in Fig 2E indicate gaze probability across participants or is it an exemplar case from each group? Is the gaze probability 1 if the participant’s eyes landed in that pixel at any time when the picture was shown & zero otherwise, with the probabilities reflecting the average across participants (and smoothing); Or does this variable get a number between [0..1] according to the time spent in each pixel (or bin of given size), per participant, normalized by the total net time?

- It seems that in the CC group, the scanning patterns of some image categories (e.g. all houses, in brown) are consistently below chance performance (AUC <0.5). How do the authors interpret this? Is there something unique about these images?

- Table 1 reports the range for some values (e.g. age, and logMAR acuity for the DC group) and not others (e.g. age at surgery, logMAR acuity for the CC group). It would be useful for this to be consistent.

- Typographic errors

• In Table 1 “unknow” should be “unknown"

• Line 125 should be “necessarily"

• Line 320 should be “depended"

• Line 331 should be “evaluated"

References

Chung, S. T. L., & Bedell, H. E. (1995). Effect of retinal image motion on visual acuity and contour interaction in congenital nystagmus. Vision Research, 35(21), 3071-3082.

De Haas, B., Iakovidis, A. L., Schwarzkopf, D. S., & Gegenfurtner, K. R. (2019). Individual differences in visual salience vary along semantic dimensions. Proceedings of the National Academy of Sciences, 116(24), 11687-11692.

Dunn, M. J., Harris, C. M., Ennis, F. A., Margrain, T. H., Woodhouse, J. M., McIlreavy, L., et al. (2019). An automated segmentation approach to calibrating infantile nystagmus waveforms. Behavior Research Methods, 51(5), 2074-2084.

Pascal, E., & Abadi, R. V. (1995). Contour interaction in the presence of congenital nystagmus. Vision Research, 35(12), 1785-1789.

Pavlidis, G. T. (1981). Do eye movements hold the key to dyslexia? Neuropsychologia, 19(1), 57-64.

Rosengren, W., Nyström, M., Hammar, B., & Stridh, M. (2020). A robust method for calibration of eye tracking data recorded during nystagmus. Behavior Research Methods, 52(1), 36-50.

## Author Response

Synthesis Statement for Author (Required):

The authors examine the eye-movement strategies used in the visual exploration of images by individuals born with dense cataracts who subsequently regained their sight. Though the visual abilities of similar individuals has been examined previously, their active exploration and eye-movement patterns were previously unknown. Since active vision is such a critical component of vision, it is a central question in sensory system development.

The cataract-recovery (CC) group are compared with a set of interesting control groups - sighted controls (SC), those with milder developmental cataracts that did not obscure sight (DC) and those with nystagmus (NC; uncontrolled eye movements of a nature similar to those in the CC group after cataract removal). The later nystagmus group are a particularly interesting inclusion given the many similarities in their active exploration with the CC group. The authors examine the exploration patterns not only through comparison between the gaze patterns of the CC and SC groups, but also with comparison to feature models of luminance contrast and higher-level saliency maps through the DeepGaze model. Their results suggest that these individuals have near-normal eye movements when freely exploring real-world images despite pervasive gaze instability. Specifically, their eye movement patterns were well accounted by predictors based on normally sighted controls and models based on low- and high-level visual features. Recent memory information also affected their scanning patterns as was the case in controls. The authors conclude that the development of efficient visual exploration patterns is not restricted by a limiting critical (or sensitive) period.

This paper studies a very unique population, allowing for studying issues in visual development that are normally intractable. The study’s main question is intriguing, and important, the analyses are creative and technically demanding, and the results are overall quite convincing. The authors have done a very thorough job using sophisticated data analysis tools, and the proper statistical techniques. They also address the issue of the image blur that the sight recovery patients suffer from, even after surgical treatment (though to a lesser extent than prior to surgery).

Reviewers have, however, identified several drawbacks, and noted the unclear description of the methodology that hindered adequate evaluation of the results and their interpretation, as detailed below.

1. Data presentation

The basic data on which the various analyses were done should be presented clearly. Figure 2E is important and should be presented and explained carefully in detail. It seems that the colors refer to the percentile of the specific pixel in the image (meaning that this is a relative measure). What about the absolute value (in terms of dwell time). This may differ significantly across groups and is currently not shown.

REPLY 1: The probability maps shown in Fig. 2 (previously Fig. 2E), as well as entropy and AUC calculations, are based on all gaze position samples which were acquired at 500 Hz. This is in contrast to a typical eye-tracking analysis in which the unit of analysis is fixations. When using fixations as the unit of analysis, the spatial distribution of the probability to fixate is indeed different to the spatial distribution of dwell time spent in different location of the image. In contrast, as we based our analysis on each and every gaze position sample, the probability to gaze a given location displayed in Fig. 2 corresponds to dwell time (with different units).

The description of Figure 2E (now Figure 2) has been complemented to be clearer and to highlight the equivalence between the probability to gaze a certain location and dwell time (see legend Fig. 2) in the present study. The spatial probability distribution shown in color has been slightly changed in the revised manuscript: In the previous manuscript each image map was normalized to the minimum and maximum probability of each image; in the revised manuscript we instead use the same scale for all images. Additionally, we added the images’ respective DG-II and ICF maps.

2. The similarity of exploration patterns between groups

The examination of eye movement parameters (as plotted in Figure 1) reveals differences between the groups, with continuous motion and greater entropy/spread in the CC and NC groups relative to the Sighted Controls, and an interesting difference between the properties of the nystagmus in CC (equivalent in all directions) and NC individuals (predominantly horizontal). However, the authors assert at several points that the visual exploration of the CC individuals is “indistinguishable from those of controls” (e.g. line 382). The measures of entropy indeed correlate between groups (p19) and the predictor map of the Sighted Controls can account for gaze positions at an above-chance level (p20), but this is not the same as the exploration patterns being indistinguishable. They are clearly similar, but there are also differences that are not being accounted for by the current measures (e.g. AUC values fall below in all cases).

REPLY 2: We reworded this part to make the conclusion less strong: Revised manuscript: “similar to those of controls” (lines 382) and “and that this dependency was similar to the one guiding controls” (line 402). We emphasize more clearly in the revised manuscript that CC individuals’ visual exploration, although predictable by the tested predictor maps, was less predictable than in SC and DC participants. However, crucially, prediction was as good as for NC individuals (for overall AUC and entropy values) (see lines 616-629). Furthermore, we followed the suggestions of the reviews and added new analyses. In particular analyzing visual exploration for different temporal phases of exploration revealed some small but significant differences between CC participants and all other groups, including NC participants (see Reply 6, 7). The discussion was adapted accordingly (see lines 616-629).

This can be seen in Fig1A (& in the movie), which are useful for appreciating the raw data. Clearly, the SC participant focuses almost solely on the parrot’s eyes; The CC participant shows partial overlap but their gaze drifts to other parts of the animal. The DC participant shows no correspondence at all (very peculiar), & the NC case is dominated by the horizontal nystagmus. Maybe this is a non-classical example or choice of participants. A few more examples may help here.

REPLY 3: We added another video and, in the extended data of Fig. 1, two additional examples.

One thing that comes to mind here is that it seems that the temporal order of the eye-movement behaviors is not considered in the analysis. Is it possible for instance that the CC individuals may scan the images in a different order than sighted controls, or that they may jump between locations more rapidly as they try to make sense of the scene, similar to the way that dyslexic readers make ‘re-fixations’ or ‘regressive eye movements’ during reading (Pavlidis, 1981)? It seems that the current analyses would be insensitive to these patterns given their dependence on spatial maps rather than temporal order.

REPLY 4: In fact, the previous analysis did not take in account neither the sequence nor possible refixations. The main reason was that we considered difficult to evaluate fixation sequences in participants with nystagmus, since it is not clear how ‘re-fixation’/‘re-gazing’ could be disentangled from the consequences of nystagmus, in which gaze drifts away from a point of interest to consecutively return to it with a saccade. In the revised manuscript we added a new analysis in which we take into account the temporal progression of exploration (see Reply 6). This analysis revealed some differences between the CC group and all other control groups.

Regardless, greater care should be taken in the statements made about the similarity between these groups. Greater discussion of the limitations of the current measures would also be ideal.

REPLY 5: A more extensive discussion of limitations of our approach and results has been added in the revised manuscript (see Replies 2, 6, 8, 13, 17).

3. Limitations of the image set

Relatedly, the image sets here are quite simple and uniform in nature - all had a single object located near the center of the image (line 161). Moreover, the stimuli were presented for 4 sec - this is a very long time for recognizing a single object. It is likely that active vision contributes to object recognition only in the first few saccades in the first 1-2 sec. It is possible that CC participants require more time for object recognition than the controls. Controls may have accomplished the task quickly, and then directed their vision to other features, which were not part of the figure object. This could have bloated the exploration, matching it more closely to that seen in the CC group. Consistent with that, the AUC is quite low (< ∼0.65) even in the SC group, suggesting that there is quite a bit of variation in the scanning patterns between SC participants. It may be worthwhile to compare performance across various time windows to test this (say comparing eye movements in the first 2 sec, vs the last 2 sec).

REPLY 6: The image set was chosen such that it allowed assessing object recognition too as well as neural correlates of object recognition (ongoing study) in visually impaired populations. This came at the cost of having less varying images (see lines 607-611/630-637 and Reply 8).

Whether lower visual acuity and prevailing nystagmus slows down object recognition in CC participants is not possible to assess with the current data. A previous study of our lab did however not find a longer latency of the N170 wave of event-related potentials to houses and faces (discussed now in lines 668-672).

AUC results are in the lower end of what has usually been reported (see e.g., Bylinskii et al., 2016; Wilming et al., 2011) likely due to at least two reasons (discussed in lines 604-615): (1) we used rather small and black and white images with one single central object. (2) We took into account each gaze sample location instead of fixation locations as done in most previous studies. This likely added redundant and less predictable segments of data to the analysis, including drifts and saccades.

We have now performed additional analysis to evaluate how exploration predictability progressed during image observation. Previous research has investigated sequential predictability by analyzing fixation order. Since this approach was not possible in the present study due to two patient groups suffering from nystagmus we used an alternative approach: AUC values were computed from data partitions obtained by dividing individuals’ gaze data in eight non-overlapping 500 ms intervals from trial start to trial end. The results of the new analyses are presented in Fig. 3d (see result section lines 426-443). For all groups, AUC values in the first time window (0 to 500 ms after image appearance) were lower than for all other time windows (Schütt et al., 2019). After 500 ms, for SC, DC and NC participants’ exploration was best predicted between 500 and 1000 ms and become less predictable in all subsequent time epochs (Onat et al., 2014; Schütt et al., 2019). In contrast, predictability of CC participants’ exploration remained the same throughout all analyzed time windows after 500 ms. This difference between CC participants and the other groups is now discussed in lines 618-629.

Another helpful approach may be to “clean” the CC & NC data by deleting all periods in which the nystagmus was present, and focus on the samples in which there was minimal movement (using a criterion similar to the offline calibration technique, or using only time points in which the velocity was within the yellow contours of the velocity map of SC & DC). If the authors are correct, after such a correction, eye movements of these two groups (CC & NC) should be more closely matched to that of the controls. Indeed, the gaze speed quantiles analysis (Ext fig 2-1) points in that direction, but it is unclear if the slowest quantile is within the speed range of the other groups (SC, & DC). The authors used a similar approach to account for the fact that the participants from the different groups have different proportions of missing data, with the ones suffering from nystagmus (CC,NC) having much greater signal loss ∼10% than the other groups (DC,SC: ∼4%). Thus, in the CC &NC groups, the actual proportion of time spent on the objects (from the total stimulus presentation time of 4 sec) was less than reported in the paper, because the times in which gaze was outside the measuring window were discarded. This should be acknowledged.

REPLY 7: In a new analysis with velocity quantiles we used 10 instead of 5 velocity quantiles but the result did not change: The slower the eyes of CC and NC participants were moving, the better their exploration patterns could be predicted. In the revised manuscript we included this analysis in the main text and displayed the results in the main Figure 3e, rather than in the extended data. We additionally added information about how CC and NC participants’ first two gaze velocity quantiles relate to the eye movement velocity during the fixation periods of SC and DC participants (Extended data Fig. 3.6). The first two velocities quantiles of most CC and NC participants fell within the range of gaze velocity during fixations of SC and DC individuals (see lines 451-453)

In a similar vein, would the CC individuals show the same similarities to controls in more complex scenes? This would surely increase the complexity of the analyses, and the variability of associated eye movements, but individuals have nonetheless been found to show reliable individual differences in their exploration in more complex image sets (De Haas, Iakovidis, Schwarzkopf, & Gegenfurtner, 2019), e.g. with some being biased more towards faces and others to text, etc. Given that individuals themselves start to vary as images become more complex, is the similarity observed here simply an artefact of the limited stimulus type?

Similarly, would the CC individuals be expected to behave the same way in more complex images with a high degree of clutter? Given that visual crowding effects are elevated in individuals with nystagmus (Chung & Bedell, 1995; Pascal & Abadi, 1995) this may yield dissociations from typical adults when they struggle to make sense of more complex scenes. Can this possibility be excluded given the current image set?

REPLY 8: Our research goal was to assess, for the first time in this population, whether CC individuals present systematic pattern of exploration as control participants. Now that we have shown that they did, the next step in fact is to test more complex images such as images with multiple objects or larger degrees of clutter (see lines 630-637 for a short discussion).

As above, greater consideration of the limitations of the study would be ideal. These may not be easy questions to address based on the current dataset, but some consideration of issues along these lines and/or acknowledgement of the simple, uncluttered, and uniform nature of the image set would help to more appropriately place the results in the context of the broader literature. The discussion makes speculations on a range of issues including development and sight recovery without really considering the nature of the representations that are meant to be similar between these groups, nor the limitations of this conclusion based on the current analyses. More critical evaluation of the results would be ideal.

REPLY 9: Limitations of our approach and results have been discussed in more detail in the revised manuscript (see Replies 2, 6, 8, 13, 17).

4. Analyses using the predictor maps

The calculation of the predictor maps using the Sighted-Control (SC) data, and the low- vs high-level feature models is clearly explained. It is not clear, however, how the data were compared with this. It would be helpful to have an example of the ICF & DG-II model expectations for one specific image, to allow intuitive comparison of the actual pattern of eye movements of each group (as shown in Fig 2e) to the two models. Currently, it is unclear how exactly the predictors from the two maps look like, and especially, what the clear differences between the two models are and where they stem from.

REPLY 10: As previous research (Kümmerer et al., 2016, 2017; Schütt et al., 2019) and our results have shown, the DG-II model is more predictable than the ICF model. Thus the DG-II model likely catches features used for the guidance of visual exploration a simple contrast model does not consider (Schütt et al., 2019). The DG-II model selects local features which serve as a basis for object classification by a deep neural network. However, the DG-II model does not segment/tag objects. Therefore, it is not necessarily apparent from just looking at the maps what these guiding features are or what the differences to other predictors are. Additionally, the DG-II model performs best when there are text or faces in the image (Kümmerer et al., 2017), which the present image dataset did not include. We added this information in lines 281-286. To better illustrate the differences and similarities between the ICF and DG-II predictor models we added examples of ICF & DG model results for the images in Fig. 2. Moreover, the grand average spatial distribution of DG-II and ICF maps across images is now shown in the extended data Fig. 2-1b.

Line 293 refers to the position “currently being tested” - does this mean that eye positions were evaluated against the predictor maps one at a time? What was the sampling rate of the recordings in this case - were they down-sampled temporally (as the example video appears to be) or was this performed at the 500Hz rate of the eye tracker? Relatedly, it is not clear how the false alarms and hits were determined (lines 295-296). If a single position at each time is being examined, is the hit rate simply the value of the current kernel/grid point, with all others set to 0, and the false alarms the opposite? Finally, is the percentage of these values taken across all time points within the presentation period, or is it averaged across an image before being entered into this analysis?

REPLY 11: We re-wrote the complete section about AUC calculation to make it clearer (see lines 294-311). It is important to emphasize that we did not actually construct receiver operating characteristic (ROC) graphs. ROC graphs can be good visualization tools to evaluate classifiers that differ markedly in their trade-off between hit rates and false alarms, but not so much for small differences in performance of the same classifier. Most importantly, to calculate the corresponding area under the curve (AUC), that gives us a single number to characterize classifier performance, it is not necessary to actually construct the ROC graph, since AUC values can be calculated easily from the Mann-Whitney U statistic as described in lines 306-311 of the revised manuscript.

In the previous description the “one” in “one currently being tested” referred to the images. That is, for each participant, gazed and non-gazed locations necessarily need to be evaluated image wise, with the non-gazed location corresponding to the locations gazed by the corresponding subject in all other images that are not the “one currently being tested”. These gazed and non-gazed values per image are then pooled together for each participant to calculate the corresponding participant AUC value (this is equivalent to take the AUC values per image and then average them, see Proof of AUC linearity in (Wilming et al., 2011)). Alternatively, for the results presented per image in Fig. 3f, the gazed and non-gazed values per participant are pooled together for each image (this is now explicitly explained in the methods, lines 309-311).

Gazed and non-gazed locations are not fixation locations, but all sampled positions by the eye-tracker, at 500 Hz. Therefore, gazed locations of an image by a given subject are the locations of all eye-tracker samples of a given participant of that image, and non-gazed location for an image are the locations of all eye-tracker samples in all other images observed by a given subject.

In order to avoid confusion, we removed the part about the construction of the ROC graph and false-alarms/hits.

The analysis was done for all gazed/non-gazed values for the complete presentation time; in the revised manuscript we additionally present analyses for different periods of exploration as explained above in Reply 6.

Videos were down sampled to 125 Hz for better visualization, this is now clarified in the corresponding captions.

The authors also compare the AUC to the shuffled case, in which the predictors were based on a different, randomly selected image from the image set. The objects, however, were located close to the center, and thus the objects in different images overlapped considerably. Shouldn’t we expect to see (on average) an AUC larger than 0.5 for the shuffled cases as well? Can the authors explain why this is not the case? This needs to be clarified (e.g. maybe show a heatmap of the “figure” overlap across all images).

REPLY 12: For the shuffled cases we would expect a value of 0.5. This is a consequence of how the AUC values are calculated, in which non-gazed locations in an image are not all (or sampled uniformly from) non-gazed locations but sampled from each participant spatial bias. This procedure is well accepted for the analysis of eye-tracking data (Bylinskii et al., 2016; Tatler et al., 2005; Wilming et al., 2011) The new extended data Fig. 2-1a displays heatmaps per group of the overall exploration bias across all images, and in Fig. 2-1b the grand average of ICF and DG-II maps across all images. These new figures allow the reader to evaluate possible biases.

Only if non-gazed locations were sampled uniformly from the images AUC values larger than 0.5 would have been expected when a strong central bias were present. Since non-gazed values are taken from individuals’ bias, the employed procedure controls for possible biases.

5. Interpretation of the results

The authors argue that the higher AUC values for the high-level DeepGaze model shows that CC individuals ’relied more on high-level visual features’ (lines 489 and 628). It is not clear that this is accurate. The DeepGaze model certainly shows impressive contextual modulations and other properties that mean it is not merely responding to low-level featural contrast, but it would also be sensitive to low-level featural contrast in some cases. It is true that the success of this model as a predictor shows that both low and high level features need to be considered in order to predict eye movement patterns, but that is not the same as saying that the observers rely more on high- than low-level features.

REPLY 13: We changed the wording according to this suggestion. In fact, we wanted to express that CC participants seem to be guided by both low- and high-level features in a similar manner as controls: We did not want to state that CC individuals necessarily relied more on high level features (see lines 470-471, 486-487, 602-603).

The authors assume that the visual exploration strategies have evolved after surgery. But this cannot be stated decisively - the patients were not completely blind before surgery. It is very possible that even in the presence of minimal perception (before surgery), oculomotor behavior was utilized to optimize vision (however limited this vision was). Indeed, as the authors state, the pre-operated patients do track light with smooth pursuit patterns (and some can count fingers from nearby). Also, if exploration strategies only evolved after surgery, one might expect that the AUC (& entropy) values would be positively correlated with the time from surgery. This is not the case (although clearly the very limited sample size makes it difficult to judge this; also please note that the data in extended Fig 2.2 are missing). The statement that (Line 701) “the remarkably preserved exploration patterns of sight-recovery individuals with a history of a transient phase of congenital patterned visual deprivation suggests that the development of visual exploration mechanisms based on low- and high-level visual features, as well as on short-term memory, do not require visual input during the initial phase following birth” is overly stated. These patients have some visual input (albeit very limited) prior to surgery. The authors ought to acknowledge this possibility in the discussion.

REPLY 14:

All patients had preserved sensitivity to light prior to surgery. In fact, this is a prerequisite for cataract surgery. The three CC individuals who met pre-surgery the criteria for “serve visually impaired” rather than blindness were the patients who had absorbed lenses and thus, had been very likely total cataracts during the first years of life (we explicitly mention this in in line 116-117 of the revised manuscript). Importantly, all CC participant showed signs of severe visual deprivation within the first weeks of life, since they all present nystagmus and the recovery of visual acuity after surgery was limited. These deficits are a direct consequence of visual deprivation within the first 8 weeks of life (Maurer et al., 2005; Röder & Kekunnaya, 2021). In this context it is important to notice that all CC individuals had systematic eye movement patters irrespective of what was noticed down as pre-surgery visual capability. Thus, we deem it as highly unlikely that CC individuals would have shown systematic eye movements pre-surgery to the images employed in the present study.

We think that if exploration strategies evolved after surgery (what we indeed think) we do not necessarily expect a correlation of AUC and entropy values with time from surgery. We cannot expect that the visual exploration strategies emerge linearly and slowly over years, similarly as we would not expect this in typical development.

6. Calculation of ‘instantaneous gaze velocity’

The authors calculate the ’instantaneous gaze velocity’ of the eye in order to assess its movement speed at any one point in time. It is not clear exactly how the equation provided gives an estimate of speed, however. The authors report (line 226-227) that “the weighted sum of the position (in screen pixels) of 6 non-consecutive gaze samples” was taken. This would certainly give an estimate of gaze instability/variability, but as reported would not give velocity. Imagine two readings. One proceeds across 6 pixels consecutively: 1 2 3 4 5 6. The other jumps in steps of ∼2 pixels before reversing: 1 3 5 6 4 2. The 6 positions are the same and would yield the same result when put into the weighted sum, but the differences between them (and thus the speed) are almost twice as large in the second example. The only way this may work is if these were not positions but rather differences/offsets from the current eye position - then the values would both have a sign (needed to give direction, as reported) and incorporate the size of the jumps between timepoints. Is that what was done? If so, the equation should be clarified on p11. If not, then this measure should not be referred to as velocity, but rather instability/variability. Relatedly, why were the positions/differences taken at n{plus minus}2, {plus minus}3, and {plus minus}4, excluding n{plus minus}?

REPLY 15: There was an error in the formula. The gaze samples in the “past” (“n-2", “n-3", “n-4”) should have had a negative sign, thus making the formula an estimate of speed. This has been corrected.

The reason for the specific form of the formula, that is, based on 6 samples and excluding n+-1 was to follow the formula used by the Eyelink eye-tracker acquisition software, which is the default velocity measure used by most researchers (that use Eyelink eye-trackers).

7. Eye-tracking calibration with nystagmus

It is extremely difficult to calibrate eye trackers with individuals whose eye movements are unstable, as for the current CC and NC groups, so this is well performed. Are these procedures similar to those reported recently for the calibration of individuals with nystagmus (Dunn et al., 2019; Rosengren, Nyström, Hammar, & Stridh, 2020)? Additionally, it is a nice touch to report the calibration error (line 184), but how was this quantified? That seems to require some ‘ground truth’ for where the subject is looking in order to be properly quantified?

REPLY 16: We used a calibration procedure that was based in simple offline manual selection of low-velocity/foveation periods, rather than in an automated procedure employed by Dun et al. 2019 and Rsengren et al, 2020. A selection of low-velocity/foveation periods was additionally performed online after the presentation of each calibration point, but this was done only to make sure at the end of calibration that the 5 points were correctly aligned (such a visual inspection is implemented in the Eyelink eye-tracker calibration procedure too). The calibration procedure has been described in more detail in the revised manuscript (lines 174-185).

Calibration error was only estimated at the center of the screen, based on the data obtained from a single center point presented after the five calibration points. This is now clearly specified in the corresponding section (lines 188-189).

8. Memory effects

The authors used a design in which in 25% of the trial pairs, the same image was repeated. They show that the entropy in the image repeat is smaller than in the first presentation. However, the two images are separated by a mere 1 sec, so making a strong case about memory from this data is somewhat misleading. Also, there is no reference in the results to the other (same category, different category) conditions. What was the reason for having them (priming effect? probably resulting in faster recognition times). A better approach might have been to show all the stimuli twice, with an intervening (fixed or variable) number of stimuli between them.

REPLY 17: The results of the same and different category conditions are now integrated in Figure 5 (now Fig. 5a). Additionally, we mention explicitly now in lines 655-657 that in this experiment the time between repetitions was shorter than in previous studies. We think it is justified to assume that the reduction of entropy is based on the short-term memory representation generated by the first exposure similarly as with longer SOAs. Since the entropy reduction was not found for category repetition, the guiding representation was more likely visual working memory rather than a semantic representation. The repetitions were included to generate a first idea whether top-down effects are considered by CC individuals for guiding eye movements at all. Our results do not necessary imply that top-down mechanisms are in general preserved (see line 655-657).

Minor comments

- With the nystagmus (NC) individuals, what form of infantile nystagmus syndrome (INS) did they have? INS can be associated with albinism, low vision, retinal dystrophies, or can be idiopathic (of no known cause). Which of these were associated with the individuals tested here? Given that the results of this group are so interesting it would be useful to be able to compare them more readily (and e.g. reported in Table 1) to the wider literature on individuals with INS.

REPLY 18: Nine of the ten INS were diagnosed with idiopathic INS and one with oculocutaneous albinism, this information has been added in line 137. As described in the table and shown in Extended data Table 1-1, NC individuals had a visual acuity that fell between the visual acuity of the congenital and developmental cataract group.

- The DC group (with early cataracts of lower severity) appear more like controls than the CC group (with dense bilateral cataracts), which is attributed to the DC group having had some vision in early life. Their acuity is also better than the CC group, however. Given that acuity can predict performance in the CC group, can we be sure the group difference is due to the early history and not simply their acuity?

REPLY 19: Our main point was that our results were compatible with visual exploration aiding object recognition in CC individuals and that active vision might have promoted in the acquisition of object knowledge (similar as it has been argued in healthy early development) (discussed in lines 676-696). Recognition performance in the CC group was overall high and we did not interpret the slightly lower level in CC than in DC individuals.

The DC group was included not only as a control for early non-congenital visual deprivation, but for the effect of surgery and, in most case, having intraocular lenses too.

- “As all CC and NC individuals suffered from nystagmus, dependent variables were defined with respect to gaze rather than fixation and saccades.” It is not clear what exactly was done. What do you mean by gaze? Did the authors use the individual sample data (@500Hz)?

REPLY 20: In fact, we used individual sample data at 500 Hz. This has been more explicitly emphasized in lines 219-228 (see Replies 1,6,11).

- “We compared the scalar value of instantaneous velocity (i.e., the magnitude of the vector composed by the horizontal and vertical components) and the overall distribution of the velocity vectors (combining magnitude and direction) between groups”. The second sentence is unclear. Velocity includes speed (magnitude) and direction (angle). What does the distribution refer to? You can have a polar plot in which both are depicted (as a probability map; similar to Fig 1c), is that what the authors mean? If so, please refer to it in the text to aid understanding.

REPLY 21: We removed this text and adapted the result section accordingly (see lines 368-374).

- Consider presenting an example (Figure 2E) of the raw data before showing the data analysis results (Fig 2a-d; i.e. reverse the order of presentation). Also, it is unclear if the visual exploration patterns (in 2E) are the group average, or an example from an individual subject. Do the colors in Fig 2E indicate gaze probability across participants or is it an exemplar case from each group? Is the gaze probability 1 if the participant’s eyes landed in that pixel at any time when the picture was shown & zero otherwise, with the probabilities reflecting the average across participants (and smoothing); Or does this variable get a number between [0..1] according to the time spent in each pixel (or bin of given size), per participant, normalized by the total net time?

REPLY 22: We changed the order of the figures. Previous Fig. 2E is now a single figure, Fig. 2, in the revised manuscript. This new Fig. 2 shows a color bar to facilitate the interpretation of the gaze probability map colors. The visual exploration patterns were constructed from the pooled data of all participants in a given group. Hence the maps indicate the probability of gazing a given location across participants. As explained above (see Reply 1,11,20), these probability maps, as well as all measures, were calculated based on individual sample data that were acquired at a constant sampling rate. Therefore, there is no difference between the spatial distribution of the probability to gaze and the spatial distribution of time spent in different locations. The probabilities depicted in Fig. 2 were constructed in the same way as it was described for the SC predictor map: Pixel-level gaze counts were spatially smoothed with a two-dimensional Gaussian unit kernel (full width at half maximum = 2 degrees) and normalized by dividing by the total count of gaze samples. We re-wrote part of the corresponding figure legend to clarify these points (see lines 956-968)

- It seems that in the CC group, the scanning patterns of some image categories (e.g. all houses, in brown) are consistently below chance performance (AUC <0.5). How do the authors interpret this? Is there something unique about these images?

REPLY 23: The images of houses consistently had lower AUC values in all groups (see Extended data Fig. 3-7). It is unclear to us why this was so, but, in general, the house images showed a close-up of a single house/building that covered most of the image (see new Video 2 for an example), whereas in the other categories, the ‘object’ size with respect to the full image was usually smaller and displayed over a more homogenous background. However, the fact that there were only seven exemplars per category makes it impossible to speculate on possible category specific differences.

- Table 1 reports the range for some values (e.g. age, and logMAR acuity for the DC group) and not others (e.g. age at surgery, logMAR acuity for the CC group). It would be useful for this to be consistent.

REPLY: Fixed.

- Typographic errors

• In Table 1 “unknow” should be “unknown"

• Line 125 should be “necessarily"

• Line 320 should be “depended"

• Line 331 should be “evaluated"

REPLY: Fixed.

-

REFERENCES

Bylinskii, Z., Judd, T., Oliva, A., Torralba, A., & Durand, F. (2016). What do different evaluation metrics tell us about saliency models? ArXiv:1604.03605 [Cs].

Kümmerer, M., Wallis, T. S. A., & Bethge, M. (2016). DeepGaze II: Reading fixations from deep features trained on object recognition. ArXiv:1610.01563 [Cs, q-Bio, Stat].

Kümmerer, M., Wallis, T. S. A., Gatys, L. A., & Bethge, M. (2017). Understanding Low- and High-Level Contributions to Fixation Prediction. 2017 IEEE International Conference on Computer Vision (ICCV), 4799-4808. https://doi.org/10.1109/ICCV.2017.513

Maurer, D., Lewis, T. L., & Mondloch, C. J. (2005). Missing sights: Consequences for visual cognitive development. Trends in Cognitive Sciences, 9(3 SPEC. ISS.), 144-151. https://doi.org/10.1016/j.tics.2005.01.006

Onat, S., Açık, A., Schumann, F., & König, P. (2014). The contributions of image content and behavioral relevancy to overt attention. PLoS ONE, 9(4), e93254. https://doi.org/10.1371/journal.pone.0093254

Röder, B., & Kekunnaya, R. (2021). Visual experience dependent plasticity in humans. Current Opinion in Neurobiology, 67, 155-162. https://doi.org/10.1016/j.conb.2020.11.011

Schütt, H. H., Rothkegel, L. O. M., Trukenbrod, H. A., Engbert, R., & Wichmann, F. A. (2019). Disentangling bottom-up versus top-down and low-level versus high-level influences on eye movements over time. Journal of Vision, 19(3), 1. https://doi.org/10.1167/19.3.1

Tatler, B. W., Baddeley, R., & Gilchrist, I. (2005). Visual correlates of fixation selection: Effects of scale and time. Vision Research, 45(5), 643-659. https://doi.org/10.1016/j.visres.2004.09.017

Wilming, N., Betz, T., Kietzmann, T. C., & König, P. (2011). Measures and Limits of Models of Fixation Selection. PloS One, 6(9), e24038. https://doi.org/10.1371/Citation
