# Supplementary material for: Active Vision in Sight Recovery Individuals with a History of Long-Lasting Congenital Blindness
Source: eNeuro. 2022 Sep 29;9(5):ENEURO.0051-22.2022. doi: 10.1523/ENEURO.0051-22.2022 (PMC9532021; doi:10.1523/ENEURO.0051-22.2022)
Supplement: Figure 1-3 — Instantaneous gaze velocity statistical result. Download Figure 1-3, DOCX file. [file enu-eN-NWR-0051-22-s31.docx]

| **Extended data Fig. 1-3.** Instantaneous gaze velocity | | | | |
| --- | --- | --- | --- | --- |
| Robust fit regression model (normal distribution, dummy coding):  speed ~ 1 + group | | | | |
| *F*_(3,38)_ = 44.1 | *p-value* = 1.87 *10^-12^ | | Adj. R-Squared = 0.76 | |
|  | | | | |
|  | Estimate | SE | t-stat | p-value |
| Intercept (CC) | 20.29 | 2.02 | 10.03 | 3.2 *10^-12^ |
| SC | -17.52 | 2.69 | -6.51 | 1.1 *10^-07^ |
| DC | -16.45 | 2.94 | -5.59 | 2.1 *10^-6^ |
| NC | 8.58 | 2.86 | 3.0 | 4.8 *10^-3^ |
|  | | | | |
| Other contrasts: |  | | | |
| SC-DC | -1.07 |  | -0.39 | 0.7 |
| SC-NC | -26.10 |  | -9.7 | 7.9 *10^-12^ |
| DC-NC | -25.03 |  | -8.51 | 2.4 *10^-10^ |
|  | | | | |
