# Supplementary material for: Active Vision in Sight Recovery Individuals with a History of Long-Lasting Congenital Blindness
Source: eNeuro. 2022 Sep 29;9(5):ENEURO.0051-22.2022. doi: 10.1523/ENEURO.0051-22.2022 (PMC9532021; doi:10.1523/ENEURO.0051-22.2022)
Supplement: Figure 3-1 — Entropy statistical result. Download Figure 3-1, DOCX file. [file enu-eN-NWR-0051-22-s30.docx]

| **Extended data Fig. 3-1.** Entropy | | | | |
| --- | --- | --- | --- | --- |
| Robust fit regression model (normal distribution, dummy coding):  entropy ~ 1 + group | | | | |
| *F*_(3,38)_ = 53.8 | *p-value* = 9.43 *10^-14^ | | Adj. R-Squared = 0.79 | |
|  | | | | |
|  | Estimate | SE | t-stat | p-value |
| Intercept (CC) | 3.4 | 0.13 | 30.79 | 1.8 *10^-28^ |
| SC | -1.67 | 0.17 | -9.67 | 8.7 *10^-12^ |
| DC | -1.16 | 2.94 | -6.14 | 3.7 *10^-7^ |
| NC | 0.21 | 2.86 | 1.13 | 0.27 |
|  | | | | |
| Other contrasts: |  | | | |
| SC-DC | -0.51 |  | -2.87 | 6.6 *10^-3^ |
| SC-NC | -1.87 |  | -10.86 | 3.2 *10^-13^ |
| DC-NC | -1.36 |  | -7.23 | 1.1 *10^-8^ |
|  | | | | |
