# Supplementary material for: Active Vision in Sight Recovery Individuals with a History of Long-Lasting Congenital Blindness
Source: eNeuro. 2022 Sep 29;9(5):ENEURO.0051-22.2022. doi: 10.1523/ENEURO.0051-22.2022 (PMC9532021; doi:10.1523/ENEURO.0051-22.2022)
Supplement: Figure 3-2 — AUC (SC predictor map) statistical result. Download Figure 3-2, DOCX file. [file enu-eN-NWR-0051-22-s29.docx]

| **Extended data Fig. 3-2.** AUC (SC predictor map) | | | | |
| --- | --- | --- | --- | --- |
| Robust fit regression model (normal distribution, dummy coding):  entropy ~ 1 + group | | | | |
| *F*_(3,38)_ = 53.8 | *p-value* = 9.43 *10^-14^ | | Adj. R-Squared = 0.79 | |
|  | | | | |
|  | Estimate | SE | t-stat | p-value |
| Intercept (CC) | 0.550 | 0.007 | 83.3 | 1.2 *10^-44^ |
| SC | 0.056 | 0.009 | 6.4 | 1.8 *10^-8^ |
| DC | 0.065 | 0.009 | 6.8 | 4.8 *10^-8^ |
| NC | 0.0008 | 0.009 | 0.09 | 0.93 |
|  | | | | |
| Other contrasts: |  | | | |
| SC-DC | -0.009 |  | -1.0 | 0.31 |
| SC-NC | 0.055 |  | 6.2 | 2.4 *10^-7^ |
| DC-NC | 0.064 |  | 6.7 | 6.3 *10^-8^ |
|  | | | | |
