# Supplementary material for: Active Vision in Sight Recovery Individuals with a History of Long-Lasting Congenital Blindness
Source: eNeuro. 2022 Sep 29;9(5):ENEURO.0051-22.2022. doi: 10.1523/ENEURO.0051-22.2022 (PMC9532021; doi:10.1523/ENEURO.0051-22.2022)
Supplement: Figure 3-4 — AUC (SC predictor) per time interval statistical result. Download Figure 3-4, DOCX file. [file enu-eN-NWR-0051-22-s28.docx]

| **Extended data Fig. 3-4.** AUC (SC predictor) per time interval | | | | | | | | | | |
| --- | --- | --- | --- | --- | --- | --- | --- | --- | --- | --- |
| Linear mixed model fit by REML. T-tests use Satterthwaite’s method (normal distribution, dummy coding):  auc ~ 1 + group*interval + (1\|subjects) | | | | | | | | | | |
|  |  | | | | |  | | |  | |
|  |  | | | | | | | | | |
|  | Estimate | | SE | | df | | | t-stat | | p-value |
| Intercept (CC) | 0.56 | | 0.008 | | 71.8 | | | 68.8 | | < 2e^-16^ |
| DC | 0.09 | | 0.011 | | 71.8 | | | 8.0 | | 1.3*10^-11^ |
| NC | 0.02 | | 0.015 | | 71.8 | | | 1.4 | | 0.16 |
| SC | 0.08 | | 0.012 | | 71.8 | | | 7.1 | | 8.6*10^-10^ |
| interval | -9.1*10^-5^ | | 0.001 | | 248 | | | -0.08 | | 0.93 |
| DC:interval | -0.006 | | 0.002 | | 248 | | | -3.7 | | 2*10^-4^ |
| NC:interval | -0.005 | | 0.002 | | 248 | | | -3.0 | | 0.003 |
| SC:interval | -0.004 | | 0.001 | | 248 | | | -2.7 | | 0.007 |
|  |  | | | | | | | | | |
|  | Random effects covariate: | | | | | | | | | |
| Intercept | 0.0004 |  | |  | | |  |  |  |  |
|  |  | | | | | | | | | |
