# Supplementary material for: Active Vision in Sight Recovery Individuals with a History of Long-Lasting Congenital Blindness
Source: eNeuro. 2022 Sep 29;9(5):ENEURO.0051-22.2022. doi: 10.1523/ENEURO.0051-22.2022 (PMC9532021; doi:10.1523/ENEURO.0051-22.2022)
Supplement: Figure 3-5 — AUC (SC predictor) per velocity quantile statistical result. Download Figure 3-5, DOCX file. [file enu-eN-NWR-0051-22-s27.docx]

| **Extended data Fig. 3-5.** AUC (SC predictor) per speed quantile | | | | | | | |
| --- | --- | --- | --- | --- | --- | --- | --- |
| Linear mixed model fit by REML. T-tests use Satterthwaite’s method (normal distribution, dummy coding):  auc ~ 1 + group*speed + (1\|subjects) | | | | | | | |
|  |  | | |  | |  | |
|  |  | | | | | | |
|  | Estimate | SE | df | | t-stat | | p-value |
| Intercept (CC) | 0.56 | 0.007 | 21 | | 83.8 | | < 2e^-16^ |
| NC | 0.005 | 0.009 | 21 | | 0.51 | | 0.61 |
| speed | -0.001 | 0.0003 | 178 | | -4.5 | | 1.1 *10^-5^ |
| NC:speed | 0.0002 | 0.001 | 178 | | 0.33 | | 0.73 |
|  |  | | | | | | |
|  | Random effects covariate: | | | | | | |
| Intercept | .0004 |  |  | |  | |  |
|  |  | | | | | | |
