# Supplementary material for: Active Vision in Sight Recovery Individuals with a History of Long-Lasting Congenital Blindness
Source: eNeuro. 2022 Sep 29;9(5):ENEURO.0051-22.2022. doi: 10.1523/ENEURO.0051-22.2022 (PMC9532021; doi:10.1523/ENEURO.0051-22.2022)
Supplement: Figure 4-1 — AUC (ICF predictor map) statistical result. Download Figure 4-1, DOCX file. [file enu-eN-NWR-0051-22-s26.docx]

| **Extended data Fig. 4-1.** AUC (ICF predictor map) | | | | |
| --- | --- | --- | --- | --- |
| Robust fit regression model (normal distribution, dummy coding):  auc ~ 1 + group | | | | |
| *F*_(3,38)_ = 14.8 | *p-value* = 1.56 *10^-6^ | | Adj. R-Squared = 0.5 | |
|  | | | | |
|  | Estimate | SE | t-stat | p-value |
| Intercept (CC) | 0.55 | 0.009 | 56.9 | 2.1 *10^-38^ |
| SC | 0.04 | 0.013 | 3.1 | 0.0039 |
| DC | 0.08 | 0.014 | 5.7 | 1.6 *10^-6^ |
| NC | -0.0001 | 0.013 | -0.01 | 0.99 |
|  | | | | |
| Other contrasts: |  | | | |
| SC-DC | -0.041 |  | -3.0 | 0.0045 |
| SC-NC | 0.04 |  | 3.1 | 0.0037 |
| DC-NC | 0.08 |  | 5.7 | 1.5 *10^-6^ |
