# Supplementary material for: Active Vision in Sight Recovery Individuals with a History of Long-Lasting Congenital Blindness
Source: eNeuro. 2022 Sep 29;9(5):ENEURO.0051-22.2022. doi: 10.1523/ENEURO.0051-22.2022 (PMC9532021; doi:10.1523/ENEURO.0051-22.2022)
Supplement: Figure 4-2 — AUC (DG-II predictor map) statistical result. Download Figure 4-2, DOCX file. [file enu-eN-NWR-0051-22-s25.docx]

| **Extended data Fig. 4-2.** AUC (DG-II predictor map) | | | | |
| --- | --- | --- | --- | --- |
| Robust fit regression model (normal distribution, dummy coding):  auc ~ 1 + group | | | | |
| *F*_(3,38)_ = 14.8 | *p-value* = 1.56 *10^-6^ | | Adj. R-Squared = 0.5 | |
|  | | | | |
|  | Estimate | SE | t-stat | p-value |
| Intercept (CC) | 0.57 | 0.01 | 53.0 | 3.1 *10^-37^ |
| SC | 0.06 | 0.014 | 4.2 | 0.0002 |
| DC | 0.09 | 0.016 | 5.6 | 1.8 *10^-6^ |
| NC | -0.009 | 0.015 | -0.6 | 0.54 |
|  | | | | |
| Other contrasts: |  | | | |
| SC-DC | -0.03 |  | -1.9 | 0.062 |
| SC-NC | 0.07 |  | 4.8 | 2.4 *10^-5^ |
| DC-NC | 0.1 |  | 6.2 | 2.8 *10^-7^ |
