# Supplementary material for: Active Vision in Sight Recovery Individuals with a History of Long-Lasting Congenital Blindness
Source: eNeuro. 2022 Sep 29;9(5):ENEURO.0051-22.2022. doi: 10.1523/ENEURO.0051-22.2022 (PMC9532021; doi:10.1523/ENEURO.0051-22.2022)
Supplement: Figure 4-3 — ICF/DG-II AUC ratio statistical result. Download Figure 4-3, DOCX file. [file enu-eN-NWR-0051-22-s24.docx]

| **Extended data Fig. 4-3.** ICF/DG-II AUC ratio | | | | |
| --- | --- | --- | --- | --- |
| Robust fit regression model (normal distribution, dummy coding):  aucRatio ~ 1 + group | | | | |
| *F*_(3,38)_ = 3.8 | *p-value* = 0.0185 | | Adj. R-Squared = 0.16 | |
|  | | | | |
|  | Estimate | SE | t-stat | p-value |
| Intercept (CC) | 0.96 | 0.009 | 97.3 | 3.4 *10^-47^ |
| SC | -0.03 | 0.013 | -2.1 | 0.045 |
| DC | -0.01 | 0.014 | -0.9 | 0.34 |
| NC | 0.015 | 0.014 | 1.0 | 0.29 |
|  | | | | |
| Other contrasts: |  | | | |
| SC-DC | -0.014 |  | -1 | 0.32 |
| SC-NC | -0.043 |  | -3.2 | 0.0027 |
| DC-NC | -0.029 |  | -2 | 0.053 |
