# Supplementary material for: Active Vision in Sight Recovery Individuals with a History of Long-Lasting Congenital Blindness
Source: eNeuro. 2022 Sep 29;9(5):ENEURO.0051-22.2022. doi: 10.1523/ENEURO.0051-22.2022 (PMC9532021; doi:10.1523/ENEURO.0051-22.2022)
Supplement: Figure 4-5 — AUC (ICF predictor) per time interval statistical result. Download Figure 4-5, DOCX file. [file enu-eN-NWR-0051-22-s22.docx]

| **Extended data Fig. 4-5.** AUC (ICF predictor) per time interval | | | | | | | | | | |
| --- | --- | --- | --- | --- | --- | --- | --- | --- | --- | --- |
| Linear mixed model fit by REML. T-tests use Satterthwaite’s method (normal distribution, dummy coding):  auc ~ 1 + group*interval + (1\|subjects) | | | | | | | | | | |
|  |  | | | | |  | | |  | |
|  |  | | | | | | | | | |
|  | Estimate | | SE | | df | | | t-stat | | p-value |
| Intercept (CC) | 0.56 | | 0.014 | | 57.9 | | | 49.2 | | < 2e^-16^ |
| DC | 0.08 | | 0.016 | | 57.9 | | | 5.0 | | 4.8*10^-6^ |
| NC | 0.008 | | 0.016 | | 57.9 | | | 0.5 | | 0.62 |
| SC | 0.05 | | 0.015 | | 57.9 | | | 3.6 | | 5.9*10^-4^ |
| interval | 1.4*10^-3^ | | 0.001 | | 248 | | | 1.2 | | 0.24 |
| DC:interval | -0.001 | | 0.002 | | 248 | | | -0.5 | | 0.6 |
| NC:interval | -0.003 | | 0.002 | | 248 | | | -1.5 | | 0.14 |
| SC:interval | -0.003 | | 0.002 | | 248 | | | -1.7 | | 0.1 |
|  |  | | | | | | | | | |
|  | Random effects covariate: | | | | | | | | | |
| Intercept | 0.0009 |  | |  | | |  |  |  |  |
|  |  | | | | | | | | | |
