# Supplementary material for: Active Vision in Sight Recovery Individuals with a History of Long-Lasting Congenital Blindness
Source: eNeuro. 2022 Sep 29;9(5):ENEURO.0051-22.2022. doi: 10.1523/ENEURO.0051-22.2022 (PMC9532021; doi:10.1523/ENEURO.0051-22.2022)
Supplement: Figure 4-6 — AUC (DG-II predictor) per time interval statistical result. Download Figure 4-6, DOCX file. [file enu-eN-NWR-0051-22-s23.docx]

| **Extended data Fig. 4-6.** AUC (DG-II predictor) per time interval | | | | | | | | | | |
| --- | --- | --- | --- | --- | --- | --- | --- | --- | --- | --- |
| Linear mixed model fit by REML. T-tests use Satterthwaite’s method (normal distribution, dummy coding):  auc ~ 1 + group*interval + (1\|subjects) | | | | | | | | | | |
|  |  | | | | |  | | |  | |
|  |  | | | | | | | | | |
|  | Estimate | | SE | | df | | | t-stat | | p-value |
| Intercept (CC) | 0.58 | | 0.012 | | 62.6 | | | 42.2 | | < 2e^-16^ |
| DC | 0.11 | | 0.018 | | 62.6 | | | 6.0 | | 8.8*10^-8^ |
| NC | 0.001 | | 0.017 | | 62.6 | | | 0.1 | | 0.94 |
| SC | 0.09 | | 0.016 | | 62.6 | | | 5.6 | | 3.6*10^-7^ |
| interval | -9.1*10^-5^ | | 0.001 | | 248 | | | 1.1 | | 0.29 |
| DC:interval | -0.004 | | 0.002 | | 248 | | | -2.1 | | 0.04 |
| NC:interval | -0.004 | | 0.002 | | 248 | | | -1.8 | | 0.06 |
| SC:interval | -0.007 | | 0.002 | | 248 | | | -3.8 | | 0.0002 |
|  |  | | | | | | | | | |
|  | Random effects covariate: | | | | | | | | | |
| Intercept | 0.001 |  | |  | | |  |  |  |  |
|  |  | | | | | | | | | |
