# Supplementary material for: Active Vision in Sight Recovery Individuals with a History of Long-Lasting Congenital Blindness
Source: eNeuro. 2022 Sep 29;9(5):ENEURO.0051-22.2022. doi: 10.1523/ENEURO.0051-22.2022 (PMC9532021; doi:10.1523/ENEURO.0051-22.2022)
Supplement: Figure 4-8 — AUC (ICF predictor map low-pass filtered, 0.5) statistical result. Download Figure 4-8, DOCX file. [file enu-eN-NWR-0051-22-s21.docx]

| **Extended data Figure 4-8.** AUC (ICF predictor low-pass 0.5 filtered) | | | | |
| --- | --- | --- | --- | --- |
| Robust fit regression model (normal distribution, dummy coding):  auc ~ 1 + group | | | | |
| *F*_(3,38)_ = 5.12 | *p-value* = 0.0045 | | Adj. R-Squared = 0.23 | |
|  | | | | |
|  | Estimate | SE | t-stat | p-value |
| Intercept (CC) | 0.97 | 0.008 | 112. | 1.6 *10^-49^ |
| SC | -0.033 | 0.012 | -2.82 | 0.007 |
| DC | -0.045 | 0.013 | -3.51 | 0.001 |
| NC | -0.013 | 0.012 | -1.07 | 0.29 |
|  | | | | |
| Other contrasts: |  | | | |
| SC-DC | 0.012 |  | 0.99 | 0.33 |
| SC-NC | -0.02 |  | -1.69 | 0.1 |
| DC-NC | -0.03 |  | -2.48 | 0.018 |
