# Supplementary material for: Active Vision in Sight Recovery Individuals with a History of Long-Lasting Congenital Blindness
Source: eNeuro. 2022 Sep 29;9(5):ENEURO.0051-22.2022. doi: 10.1523/ENEURO.0051-22.2022 (PMC9532021; doi:10.1523/ENEURO.0051-22.2022)
Supplement: Figure 4-9 — AUC (DG-II predictor map low pass 0.5 filtered, 0.5) statistical result. Download Figure 4-9, DOCX file. [file enu-eN-NWR-0051-22-s20.docx]

| **Extended data Figure 4-9.** AUC (DG-II predictor low-pass 0.5 filtered) | | | | |
| --- | --- | --- | --- | --- |
| Robust fit regression model (normal distribution, dummy coding):  auc ~ 1 + group | | | | |
| *F*_(3,38)_ = 7.8 | *p-value* = 3.5 *10^-3^ | | Adj. R-Squared = 0.33 | |
|  | | | | |
|  | Estimate | SE | t-stat | p-value |
| Intercept (CC) | 0.98 | 0.007 | 138.9 | 4.8 *10^-53^ |
| SC | -0.03 | 0.009 | -3.13 | 0.003 |
| DC | -0.04 | 0.01 | -3.9 | 3.8 *10^-4^ |
| NC | -0.003 | 0.01 | -0.26 | 0.79 |
|  | | | | |
| Other contrasts: |  | | | |
| SC-DC | 0.011 |  | 1.1 | 0.03 |
| SC-NC | -0.03 |  | -2.8 | 6.9 *10^-3^ |
| DC-NC | -0.04 |  | -3.6 | 8.05 *10^-4^ |
