# Supplementary material for: Active Vision in Sight Recovery Individuals with a History of Long-Lasting Congenital Blindness
Source: eNeuro. 2022 Sep 29;9(5):ENEURO.0051-22.2022. doi: 10.1523/ENEURO.0051-22.2022 (PMC9532021; doi:10.1523/ENEURO.0051-22.2022)
Supplement: Figure 5-1 — Entropy values for the first versus second image of a pair of identical images statistical result. Download Figure 5-1, DOCX file. [file enu-eN-NWR-0051-22-s19.docx]

| **Extended data Figure 5-1.** Entropy values for the first vs. second image of a pair of identical images | | | | | | | |
| --- | --- | --- | --- | --- | --- | --- | --- |
| Linear mixed model fit by REML. t-tests use Satterthwaite's method (normal distribution, effect coding): entropy ~ group*pair + (1\|subjects) | | | | | | | |
|  |  | | |  |  |  |  |
|  | |  | | | | | |
|  | | Estimate | SE | | df | t-stat | p-value |
| Intercept | | 3.3 | 0.06 | | 37.9 | 53.1 | < 2*10^-16^ |
| CC | | 0.63 | 0.11 | | 37.9 | 5.8 | 1 *10^-6^ |
| DC | | -0.54 | 0.11 | | 37.9 | -4.8 | 2.8 *10^-5^ |
| NC | | 0.87 | 0.11 | | 37.9 | 7.9 | 1.3 *10^-9^ |
| Pair1 | | 0.07 | 0.03 | | 38 | 2.8 | 8.4 *10^-3^ |
| CC:pair1 | | 0.004 | 0.05 | | 38 | 0.1 | 0.93 |
| DC:pair1 | | 0.03 | 0.05 | | 38 | 0.7 | 0.5 |
| NC:pair1 | | -0.08 | 0.05 | | 38 | -1.6 | 0.11 |
|  | |  | | | | | |
|  | | Random effects covariate: | | | | | |
| Intercept | | 0.36 |  | |  |  |  |
