# Supplementary material for: Active Vision in Sight Recovery Individuals with a History of Long-Lasting Congenital Blindness
Source: eNeuro. 2022 Sep 29;9(5):ENEURO.0051-22.2022. doi: 10.1523/ENEURO.0051-22.2022 (PMC9532021; doi:10.1523/ENEURO.0051-22.2022)
Supplement: Figure 5-2 — Entropy values for the first versus second image of a pair of images from same category statistical result. Download Figure 5-2, DOCX file. [file enu-eN-NWR-0051-22-s18.docx]

| **Extended data Figure 5-2.** Entropy values for the first vs. second image of a pair of images from same category | | | | | | | |
| --- | --- | --- | --- | --- | --- | --- | --- |
| Linear mixed model fit by REML. t-tests use Satterthwaite's method (normal distribution, effect coding): entropy ~ group*pair + (1\|subjects) | | | | | | | |
|  |  | | |  |  |  |  |
|  | |  | | | | | |
|  | | Estimate | SE | | df | t-stat | p-value |
| Intercept | | 3.3 | 0.06 | | 37.9 | 53.5 | < 2*10^-16^ |
| CC | | 0.69 | 0.11 | | 37.9 | 6.4 | 1.5 *10^-7^ |
| DC | | -0.48 | 0.11 | | 37.9 | -4.3 | 1 *10^-3^ |
| NC | | 0.83 | 0.11 | | 37.9 | 7.7 | 3.1 *10^-9^ |
| Pair1 | | 0.02 | 0.02 | | 38 | 0.9 | 0.36 |
| CC:pair1 | | -0.007 | 0.03 | | 38 | -0.2 | 0.85 |
| DC:pair1 | | 0.015 | 0.04 | | 38 | 0.4 | 0.68 |
| NC:pair1 | | -0.023 | 0.03 | | 38 | -0.6 | 0.51 |
|  | |  | | | | | |
|  | | Random effects: | | | | | |
| Intercept | | 0.37 |  | |  |  |  |
