# Supplementary material for: Active Vision in Sight Recovery Individuals with a History of Long-Lasting Congenital Blindness
Source: eNeuro. 2022 Sep 29;9(5):ENEURO.0051-22.2022. doi: 10.1523/ENEURO.0051-22.2022 (PMC9532021; doi:10.1523/ENEURO.0051-22.2022)
Supplement: Figure 5-4 — Performance for each group statistical result. Download Figure 5-4, DOCX file. [file enu-eN-NWR-0051-22-s16.docx]

| **Extended data Fig. 5-4.** Performance for each group | | | | |
| --- | --- | --- | --- | --- |
| Generalized linear regression model (binomial distribution, dummy coding):  logit(# correct) ~ 1 + group | | | | |
| *χ^2^*_(26)_ = 97.5 | *p-value* = 6.672 *10^-22^ | | | |
|  | | | | |
|  | Estimate | SE | t-stat | p-value |
| Intercept (CC) | 1.68 | 0.12 | 14.46 | 2 *10^-47^ |
| DC | 1.67 | 0.27 | 6.14 | 8.0 *10^-10^ |
| NC | 2.43 | 0.35 | 6.84 | 7.5 *10^-12^ |
|  | | | | |
| Other contrasts: |  | | | |
| DC-NC | -0.76 |  | -1.82 | 0.08 |
