# Supplementary material for: Active Vision in Sight Recovery Individuals with a History of Long-Lasting Congenital Blindness
Source: eNeuro. 2022 Sep 29;9(5):ENEURO.0051-22.2022. doi: 10.1523/ENEURO.0051-22.2022 (PMC9532021; doi:10.1523/ENEURO.0051-22.2022)
Supplement: Figure 5-5 — CC participants’ performance statistical result. Download Figure 5-5, DOCX file. [file enu-eN-NWR-0051-22-s15.docx]

| **Extended data Fig. 5-5.** CC participants’ performance | | | | | | |
| --- | --- | --- | --- | --- | --- | --- |
| Generalized linear regression model (binomial distribution, z-scored predictors):  logit(# correct) ~ 1 + AUC + logMAR | | | | | | |
| *AIC* = 78.8 |  | | | | | |
|  | | | | | | |
|  | Estimate | SE | Z value | | p-value | |
| Intercept (CC) | 2.5 | 0.23 | 10.9 | | < 2 *10^-16^ | |
| AUC | 1.1 | 0.21 | 5.4 | | 7.6 *10^-8^ | |
| logMAR | -1.2 | 0.15 | 6.-8.2 | | < 2 *10^-16^ | |
|  | | | | | | |
| Model comparison | AIC | R^2^ Tjur | |  | |  |
| AUC+logMAR | 78.89 | 0.21 | |  | |  |
| logMAR | 116.3 | 0.12 | |  | |  |
| AUC | 163.3 | 0.06 | |  | |  |
