# Supplementary material for: Active Vision in Sight Recovery Individuals with a History of Long-Lasting Congenital Blindness
Source: eNeuro. 2022 Sep 29;9(5):ENEURO.0051-22.2022. doi: 10.1523/ENEURO.0051-22.2022 (PMC9532021; doi:10.1523/ENEURO.0051-22.2022)
Supplement: Figure 5-7 — CC participants’ performance and age statistical result. Download Figure 5-7, DOCX file. [file enu-eN-NWR-0051-22-s14.docx]

| **Extended data Fig. 5-7.** CC participants’ performance and age | | | | |
| --- | --- | --- | --- | --- |
| Generalized linear regression model (binomial distribution, z-scored predictors):  logit(# correct) ~ 1 + age at test | | | | |
| *AIC* = 195.6 |  | | | |
|  | | | | |
|  | Estimate | SE | Z value | p-value |
| Intercept (CC) | 1.6 | 0.11 | 14.4 | < 2 *10^-16^ |
| Age at test | 0.18 | 0.13 | 1.3 | 0.17 |
